# The impact of crystal phase transition on the hardness and structure of kidney stones

**DOI:** 10.1007/s00240-024-01556-5

**Published:** 2024-04-02

**Authors:** Uta Michibata, Mihoko Maruyama, Yutaro Tanaka, Masashi Yoshimura, Hiroshi Y. Yoshikawa, Kazufumi Takano, Yoshihiro Furukawa, Koichi Momma, Rie Tajiri, Kazumi Taguchi, Shuzo Hamamoto, Atsushi Okada, Kenjiro Kohri, Takahiro Yasui, Shigeyoshi Usami, Masayuki Imanishi, Yusuke Mori

**Affiliations:** 1 Graduate School of Engineering, Osaka University, 2-1, Yamadaoka, Suita, 565-0871 Japan; 2 Graduate School of Life and Environmental Sciences, Kyoto Prefectural University, 1-5, Hangi-cho, Shimogamo, Sakyo-ku, Kyoto, 606-8522 Japan; 3 Department of Nephro-urology, Graduate School of Medical Sciences, Nagoya City University, 1-Kawasumi, Mizuho- cho, Mizuho-Ku, Nagoya, 467-8601 Japan; 4 Institute of Laser Engineering, Osaka University, 2-6, Yamadaoka, Suita City, 565-0871 Osaka Japan; 5 Department of Earth Science, Tohoku University, 6-3 Aza-Aoba, Aramaki, Aoba-ku, Sendai, 980-8578 Japan; 6 National Museum of Nature and Science, 4-1-1 Amakubo, Tsukuba, 305-0005 Japan; 7 Tajiri Thin Section Laboratory, 3-1-11 Sannose, Higashiosaka, 577-0849 Osaka Japan

**Keywords:** Kidney stones, Calcium oxalate, Microstructure, X-ray micro-CT, Solution-mediated phase transition, Extracorporeal shock wave lithotripter

## Abstract

Calcium oxalate kidney stones, the most prevalent type of kidney stones, undergo a multi-step process of crystal nucleation, growth, aggregation, and secondary transition. The secondary transition has been rather overlooked, and thus, the effects on the disease and the underlying mechanism remain unclear. Here, we show, by periodic micro-CT images of human kidney stones in an ex vivo incubation experiment, that the growth of porous aggregates of calcium oxalate dihydrate (COD) crystals triggers the hardening of the kidney stones that causes difficulty in lithotripsy of kidney stone disease in the secondary transition. This hardening was caused by the internal nucleation and growth of precise calcium oxalate monohydrate (COM) crystals from isolated urine in which the calcium oxalate concentrations decreased by the growth of COD in closed grain boundaries of COD aggregate kidney stones. Reducing the calcium oxalate concentrations in urine is regarded as a typical approach for avoiding the recurrence. However, our results revealed that the decrease of the concentrations in closed microenvironments conversely promotes the transition of the COD aggregates into hard COM aggregates. We anticipate that the suppression of the secondary transition has the potential to manage the deterioration of kidney stone disease.

**Supplementary Information:**

The online version contains supplementary material available at 10.1007/s00240-024-01556-5.

## Introduction

Biomineralization is an essential process for diverse living organisms to form hard materials inside and outside of the living system, such as shells, pearls, exoskeletons, crustaceans, bones, and teeth [1–3]. These hard materials often have excellent physical properties due to their precise crystal textures [4, 5]. This excellent hardness supports the biological activities of many living organisms, including humans. However, biomineralization often causes pathological problems for humans [6–9]. One of the most common diseases related to biomineralization is kidney stone disease, in which stones form in the urine tract. The number of patients with the disease is increasing, and the recurrence rate within five years after diagnosis is exceptionally high at ≥ 45%. The condition is serious because repeated recurrences can lead to deterioration of renal function, which is ultimately life-threatening [10, 11]. Even today, the most effective way to prevent stone recurrence is to “drink plenty of water,” and this has not changed for about 2,000 years [12].

Calcium oxalate stones, which contain about 90% of crystalline components and remaining organic matrix such as proteins [13, 14] account for about 80% of kidney stones [15]. This type of kidney stone includes two phases of calcium oxalate: calcium oxalate monohydrate (COM; a stable phase), and calcium oxalate dihydrate (COD; a metastable phase) (Supplementary Fig. 1).

Many types of investigations, including microstructure observation of kidney stones collected by surgery and laboratory experiments to demonstrate kidney stone formation, revealed that kidney stones typically form through crystal nucleation, growth, aggregation, and secondary processes in kidney stones [16–19]. The process of crystal nucleation, growth, and aggregation have been extensively investigated because these processes form and increase the size of kidney stones. The secondary processes have been rather overlooked. Based on microstructure observation of calcium oxalate kidney stones, it has been suggested that a part of COM was formed by the phase transition from COD in the secondary processes [9, 19–21]. However, how this transition is triggered and how this transition affects the pathological treatment of kidney stone disease are unknown. We investigated the transitions of crystal phases by acquiring periodic micro-CT images in an ex vivo incubation experiment of human kidney stones.

## Materials and methods

### Ethics statement

The research project presented in this paper was approved by the institutional review board of the graduate school of medicine, Nagoya City university (No.60-19-0185). All methods were carried out in accordance with the relevant guidelines and regulations. Written informed consent from all subjects was obtained according to procedures approved by the ethical committee board.

The general flow of the phase transition experiment is outlined in the Supporting data (Supplementary Fig. 2).

### Materials

Sodium chloride (purity 99.5%), calcium chloride dihydrate (purity 99.9%), and sodium oxalate (purity 99.5%) were procured from Fujifilm Wako Pure Chemicals Co. All reagents were used without further purification. Deionized water, with a resistivity of 18.2 MΩ∙cm, was prepared by Urupure (Komatsu Electronics Corporation) and employed in the experiments. COD stones were sourced from patient samples (Supplementary Fig. 2a). This stone spontaneously passed through in the urine of a male patient (Sample1; 60-years old with a history of kidney stone disease). Ethical protocols were strictly followed in the handling of the samples for the experiment.

### Solution preparation

Stock solutions were prepared for the following compositions: 150 mM NaCl solution, 10 mM CaCl_2_ solution, and a mixture of 150 mM NaCl solution and 10 mM CaC_2_O_4_ solution. These solutions were combined in a 20 ml glass vial to create a supersaturated solution, with the following amounts of each solution used: 4.5 ml of NaCl solution, 5.0 ml of NaCl and CaCl_2_ solution, and 0.5 ml of NaCl and CaC_2_O_4_ solution. The prepared solutions were incubated at room temperature (20°C) for 24 h, during which calcium oxalate nucleation occurred. Following the incubation period, the calcium oxalate solution and crystals in the vial were transferred to a 15 ml centrifuge tube (TPP) and centrifuged at 5000 rpm for 5 min using a TOMY MX-301 centrifuge. Subsequently, the supernatant solution was carefully transferred to a 10 ml syringe (Thermo) and filtered (25HP020AN, ADVANTEC) to prevent precipitated crystals from entering the bottom of the centrifuge tube. Finally, the calcium oxalate solution was transferred to a 20 ml glass vial. The entire preparation process was conducted at room temperature (20°C).

The solution prepared in the above process reproduced only the representative ion concentrations present in urine and did not contain any proteins or other organic matter.

### Induced phase transition

The COD stone (Sample 1) was placed in a glass vial containing the prepared calcium oxalate solution, and the vial was subsequently placed in an incubator set at 37°C. The solution was incubated with gentle agitation at 70 rpm using a rotary shaker (SH-BD04, Ivy Corporation). After one week, the stone was carefully removed from the solution and promptly rinsed with 95% ethanol. The stone was evaluated after appropriate washing. The detailed evaluation process is described below. Following the evaluation, the COD stone was once again immersed in a freshly prepared calcium oxalate solution. After an additional week, the stone was retrieved and subjected to further evaluation. During the experiment, the supersaturation of calcium oxalate changed (Supplementary Fig. 3).

### Balk evaluation

The surface and color of the stone were observed by a digital microscope (Dino-Line, Anmo). For a more detailed analysis of the surface structure, scanning electron microscopy (SEM: VHX-D510N, KEYENCE) was employed (Supplementary Fig. 2b). We roughly determined the crystal phases of the COD stone surface by comparison with the observed crystal morphology, the previously reported COM and COD morphologies [22, 23], and the typical morphology calculated with VESTA56 [24]. The three-dimensional crystal structure inside the stone was observed by X-ray micro-CT (ScanXmate-L090T, Com Scan Tecno Co.) (Supplementary Fig. 2c). The measurement conditions were as follows: tube voltage: 50 kV, tube current: 160 µA, and resolution: 13.256 μm/pixel. The distribution of COM and COD within the stones was determined from the structure and CT values [25].

### Thin section evaluation

The stone was processed into thin sections with 20–30 μm thickness [26] (Supplementary Fig. 2d, e). Microstructures of crystals were observed with a polarizing microscope (OPTIPHOT2-POL, Nikon). The crystal phases in the sample were roughly identified from the extinction of light when the stage was rotated. The further detailed components were decided by Raman spectra taken by a Raman spectrometer (RAMANtouch, Nanophoton) (Supplementary Fig. 2f). The measurement conditions of the Raman spectrometer are shown below. The laser used for the measurements had a wavelength of 785 nm, a laser power of 1.50 ± 0.25 mW, and the wavenumber ranged from 600 to 1600 cm^− 1^. We used a grating of 600 gr/mm. All measurement were performed at room temperature. COD, COM, and apatite were identified by referring to the specific peaks derived from the bonding of each material (Supplementary Fig. 2f). COM was identified by reference to the 894 cm^− 1^ peak derived from the C-C bond and the 1462 and 1488 cm^− 1^ peaks derived from the C = O bond. COD was identified by the peak at 911 cm^− 1^ derived from the C-C bond and the peak at 1478 cm^-1^ derived from the C = O bond. Apatite was identified by reference to the peak at 960 cm^− 1^ derived from the bond [27]. Multiple immunofluorescence staining was performed to visualize the protein distribution of the stones and was observed with Auto-Fluorescence Microscopy (Nikon A1R) [28].

### Stone crushing

Experimental stone crushing was performed using an extracorporeal shock wave lithotripter (Dornier MedTech, Dornier Gemini) (Supplementary Fig. 4a). A water tank was positioned above the extracorporeal shock wave lithotripter (Supplementary Fig. 4b). A 2 mm mesh cage was attached to the top of the tank, and stones were placed inside the cage. The shock wave intensity of the extracorporeal shock wave lithotripter was set to Lev.1 (Supplementary Fig. 4b) and the frequency to 60 Hz, and shock waves were applied to the stones 100 times. Shock waveform data was acquired with a piezoelectric sensor (119M31, PCB PIEZOTRONICS) and output with an oscilloscope (TDS 2022 C, Tektronix). The acquired data were obtained by substituting *C*_*p*_=21.8 [pF], *C*_*o*_=333 [pF] and *sens* = 0.311 [pC/PSI] into the power to pressure calibration Eq. (1).

1 $$P=\frac{sens}{{C}_{p}+{C}_{o}} [\text{P}\text{S}\text{I}/\text{V}]=\frac{sens [\text{p}\text{C}/\text{P}\text{S}\text{I}]}{\left({C}_{p} \left[\text{p}\text{F}\right]+{C}_{o} \left[\text{p}\text{F}\right]\right)\times 0.145 \left[\text{P}\text{S}\text{I}\right]} [\text{M}\text{P}\text{a}/\text{V}]$$

## Results and discussion

### Observation of the kidney stone surface

**Fig. 1:**
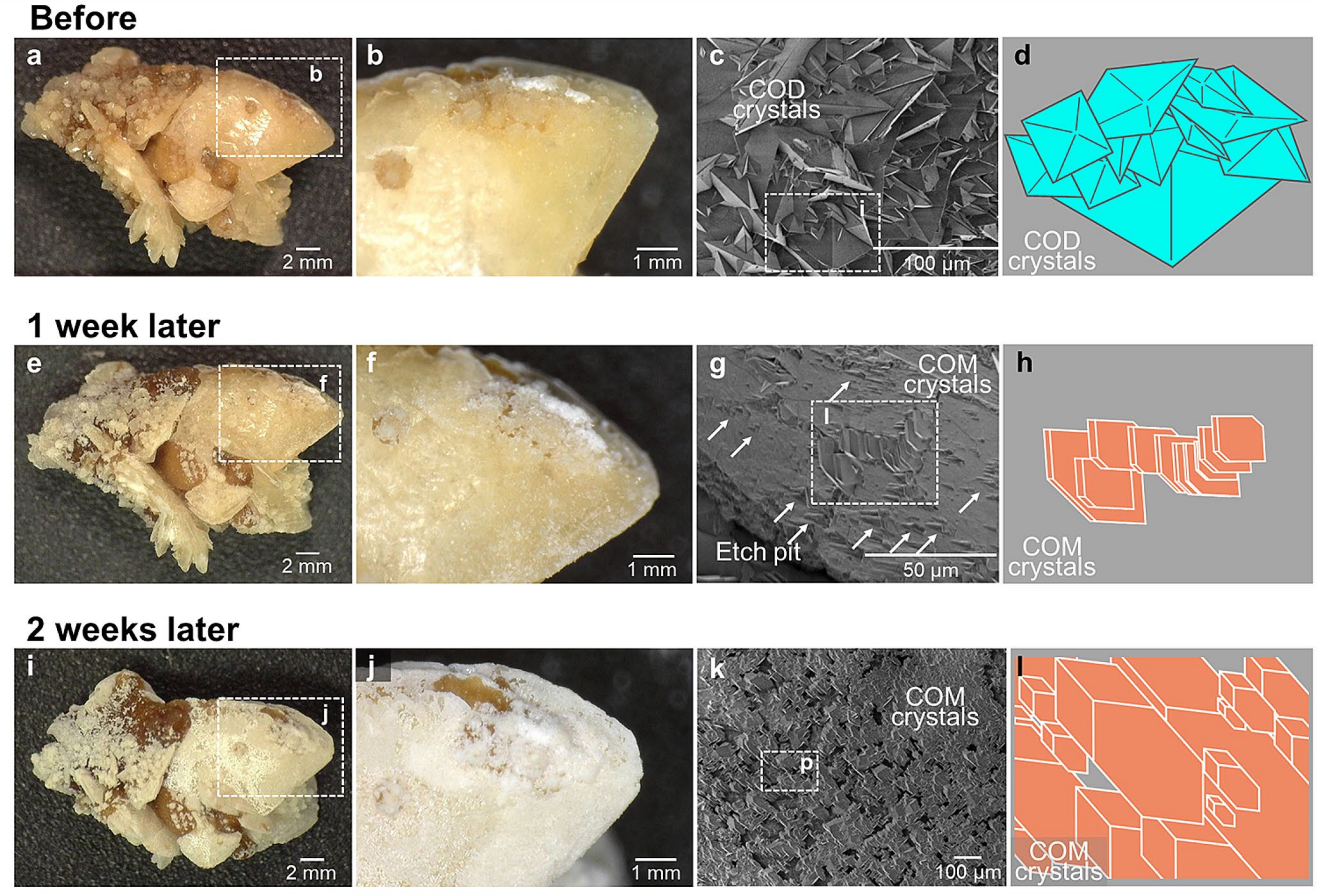
Changes in the COD stone (Sample 1) surface during the experiment. (**a**) The stereoscopic image of the COD stone at the start of the experiment. The stone is formed via the aggregation of several COD crystal. (**b**) The enlarged image of the white dotted box area in (**a**). (**c**) The SEM image. (**d**)A model diagram of the white dotted box area in (**c**). (**e**) The stereoscopic image of the COD stone one week after the start of the experiment. Etch-pits formed due to dissolution of the COD crystal surface. (**f**) The enlarged image of the white dotted box area in (**e**). (**g**) The SEM image. (**h**) A model diagram of the white dotted box area in (**g**). (**i**) The stereoscopic image of the COD stone two weeks after the start of the experiment. The stone surface was covered with COM crystals. (**j**) The enlarged image of the white dotted box area in (**i**). (**k**) The SEM image. (**l**) A model diagram of the white dotted box area in (**k**)

A kidney stone (Sample 1) is composed of several aggregates of typical COD crystals (Fig. 1a, Supplementary Fig. 1b, d) [29–31], and the COD crystals forming Sample 1 are found to have some transparency before the phase transition experiment (Fig. 1b). Scanning electron microscope (SEM) observation of the stone surface shows that the COD crystals are randomly aggregated regardless of their planes and orientations, and that the crystals vary in size (Fig. 1c, d). The surface of a few mm-sized COD crystals was covered with COD crystals of several tens µm in size (Supplementary Fig. 5a, b).

When the COD stone was immersed in supersaturated calcium oxalate solution, their surfaces became whiter, and the roughness increased as time progressed (Fig. 1e, f). Detailed observation of the stone surface after one week of immersion by SEM revealed many etch pits on the COD crystal surfaces, which were caused by dissolution (Fig. 1g, Supplementary Fig. 5c, d). At the same time, multiple COM crystals of several µm in size nucleated and grew on the surface of the COD crystals with the *b*-axis facing outward (Fig. 1g, h). After immersion in the solution for another week, the entire stone surface was covered with fine COM crystals (Fig. 1i, j). The COM crystals were aligned and oriented on the surface (Fig. 1k, l, Supplementary Fig. 5e, f).

### Changes inside the kidney stone

**Fig. 2:**
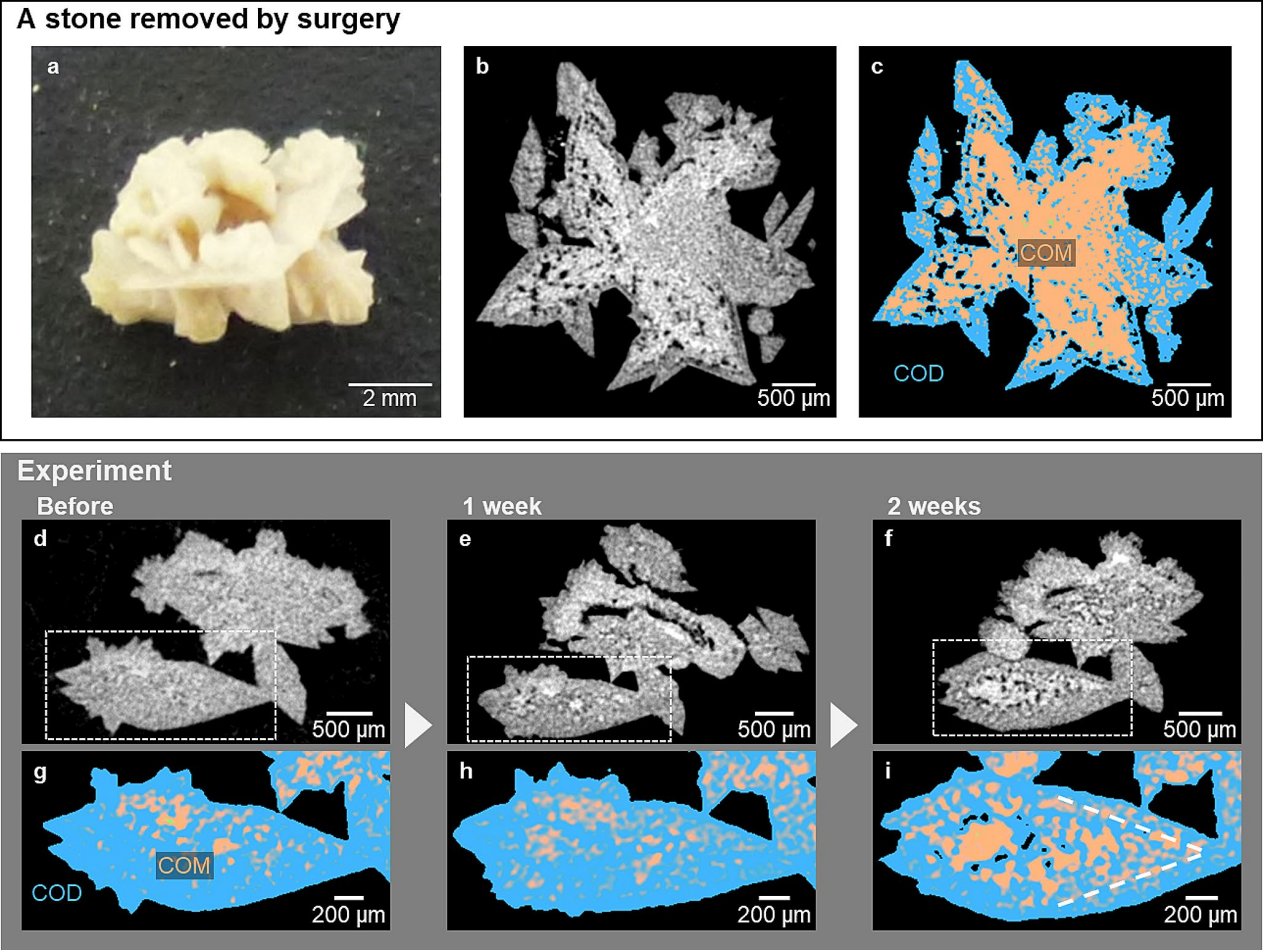
Interior of a calcium oxalate stone removed from a patient﻿ (Sample 2) and changes in the COD stone﻿ (Sample 1) interior during in the experiment. (**a**) A stereoscopic image of a commonly reported COD stone (Sample 2). (**b**) The micro-CT image of the COD stone shows in Fig. 2(**a**) (Sample 2﻿). (**c**)An image with the CT score originating from COD in blue and that originating from COM in orange. The micro-CT image of Sample 2﻿ shows a difference in the density of components between the inside and the outside of the stone. The exterior stone is relatively low-density components (COD), and the interior is higher densities components (COM). (**d**) The micro-CT image of Sample 1 at the start of the experiment. (**e**) The micro-CT image of Sample 1﻿ after one week. (**f**) The micro-CT image ofSample 1﻿ after two weeks. (**g**) The mapping ofSample 1﻿ at the start of the experiment. The COM region and COD region determined based on the CT score. (**h**) The mapping ofSample 1﻿ after one week. (**i**) The mapping ofSample 1﻿ after two weeks. The inside of the COD stone has developed voids and a mosaic-like structure, and the CT score indicates that the interior of the COD stone has changed to consist of mainly COM crystals

While the macrotextures appeared on the stone surface as described above, greater changes were observed in the inside of the stone. An example of a surgically removed stone (Sample 2) shown in Fig. 2a has COD crystal morphology, but most of the interior was filled with COM. Micro-CT images show that COM crystals with many voids exist inside of the COD crystals (Fig. 2b, c). Such stones have been seen frequently.

Another observed stone shown in Fig. 2d (Sample 1) also had COD crystal morphology, but originally composed mostly of COD with < 100 μm several COM domains in the inside of the COD single crystal. This crystal had almost no voids in the inside the crystal before the incubation experiment (Fig. 2d, g). It is common to find stones with COD in periphery region and COM in the center region [9, 21, 32–34]. By the incubation of this stone in the simulated urine solution for two weeks, periodic micro-CT images show the increase in the abundances and the sizes of the COM domains and the abundance of void areas in the inside of the COD. Finally, the incubated kidney stone (Sample 1) became closer to another kidney stone (Sample 2) with COD morphology filled with COM and voids (Fig. 2e, f, h, i).

### Evaluation of thin section

**Fig. 3:**
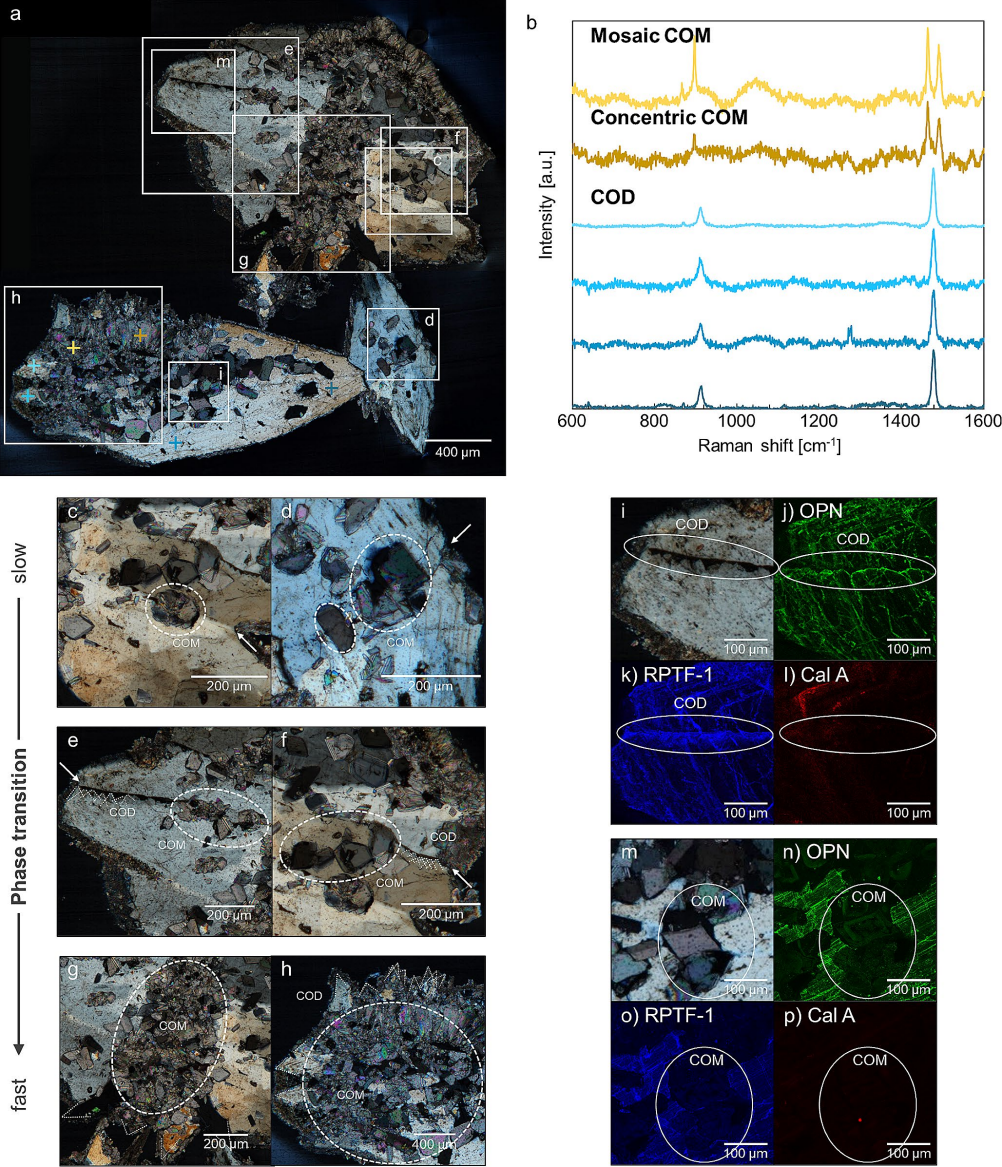
Analysis of a COD stone (Sample 1)﻿ section after the experiment. (**a**) Cross-Nicol image of a thin section of Sample 1. The degree of phase transition differs depending on regions. (**b**) Results of Raman spectroscopy. The crossed points in the cross-Nicol image were measured. (**c, d**) Enlarged cross-Nicol image of a thin section. The phase transition is less pronounced inside narrow grain boundaries or cracks. (**e, f)** Enlarged cross-Nicol image of a thin section. As the grain boundaries become wider, COD crystals nucleate within the grain boundary and the phase transition proceeds further inside. (**g**, **h**) Enlarged cross-Nicol image of a thin section. Phase transition is in full progress. (**i**) Cross-Nicol image of COD structure. (**j**) Mapping of OPN. (**k**) Mapping of RPTF-1. (**l**) Mapping of Cal A. (**m**) Cross-Nicol image of mosaic COM structure. (**n**) Mapping of OPN. (**o**) Mapping of RPTF-1. (**p**) Mapping of Cal A

In order to observe the internal structure in more detail, a thin section of Sample 1 was analyzed with a polarized microscope and Raman spectrometer (Fig. 3a, b, Supplementary Fig. 6). COM crystals formed by phase transition inside COD crystals. They present typical COM mosaic structures (Fig. 3c, d, e, f, g, h). The outline angle of the mosaic COM structures (orange dashed line in Supplementary Fig. 6b, e) ranged from 40 to 45°, which was similar to the face angle of COD crystals (Supplementary Fig. 1b, Supplementary Fig. 6b-f). This phenomenon is identical to “pseudocrystals” in natural minerals such as quartz [35]. Pseudocrystals occur when the original mineral gradually transforms into a different crystalline phase while maintaining its external shape.

Comparison of multi-IF staining fluorescence intensities of a thin section of Sample 1 shows that the calcium-binding proteins, osteopontin (OPN) and renal prothrombin fragment 1 (RPTF-1), are abundantly distributed in the COD crystals (Fig. 3i, j, k, m, Supplementary Fig. 7a, b, c, d) and the concentric COM structure (Supplementary Fig. 7e, f, g, h). Furthermore, apparent protein striations appeared in these structures. In contrast, in the mosaic COM structure (Fig. 3m), the relative protein distribution is low, and proteins distributed uniformly (Fig. 3n, o, p). Calgranulin A (Cal A) is faintly distributed in COD and COM structure (Fig. 3l, p). OPN and RPTF-1 proteins which can be incorporated into the crystals interior due to their strong affinity to the crystals [28]. OPN and RPTF-1 were partially exposed to the solution during the phase transition, and then reincorporated into the newly crystallized COM crystals. Note that no protein was added to the solution in this phase transition experiment. Since some of the proteins leaked into the solution during this process, the relative amount of protein inside the COM crystals decreased. The distribution of proteins in the stones after the phase transition experiment strongly supports that the mosaic COM structure did form in the experiment by the mechanism of solution-mediated phase transition.

### Crystal defects affect phase transition

Upon close observation of a thin section of Sample 1, it becomes evident that the extent of phase transition within COD crystals is not uniform. The observed regions are characterized by a coexistence of areas exhibiting minimal phase transition (Fig. 3c, d) and areas where phase transition progressed remarkably (Fig. 3g, h). We posit that this variation in phase transition extent is intrinsically linked to the rate of phase transition.

When classified by phase transition rate, we found approximately three patterns. Firstly, in regions classified as slow phase transition rate, notably narrow grain boundaries or cracks are observable (Fig. 3c, d). Between these grain boundaries or cracks, COM crystals nucleate and impede solution penetration. Secondly, in regions classified as medium phase transition rate, wider grain boundaries are observed compared to those previously mentioned (Fig. 3e, f). Along these grain boundaries, the growth of COD crystals is apparent. The evidence of these grown crystals as COD is substantiated by their crystallographic facet orientations and the multi-IF staining outcomes. We reported the selective attachment of RPTF-1 to the {101} face of COD crystals [28]. Between the grain boundaries in our results, the fluorescence intensity of RPTF-1 is notably heightened (Fig. 3o). As COD crystals grow to obstruct grain boundaries, phase transition to COM crystals progresses within the inner regions. Notably, this phase transition rate exceeds that observed within the narrower grain boundaries. Finally, in regions classified by the fastest phase transition rate, aggregations of numerous COD crystals were observed (Fig. 3g, h). There are a large number of grain boundaries in such the aggregations.

These observations substantiate the assertion that phase transition rate accelerates concomitantly with wider or a large number of grain boundaries. This thus implies that crystal defects, such as grain boundaries and cracks, significantly influence the phase transition phenomenon.

### Mechanism of phase transition

**Fig. 4:**
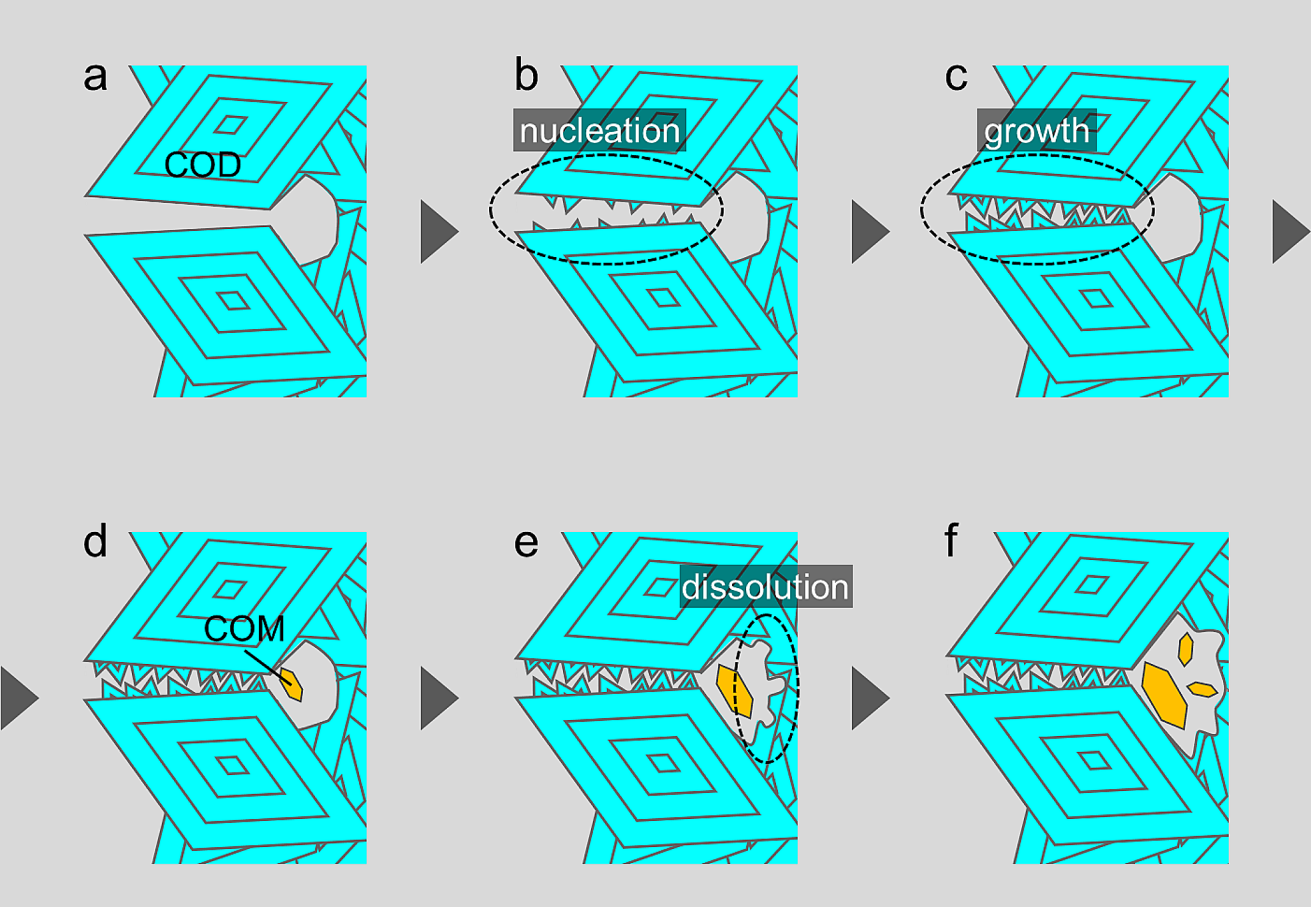
The mechanism of phase transition inside COD stones. (**a**) Grain boundaries composed of COD crystals. (**b**) Nucleation of crystals between the grain boundary. (**c**) Growth of crystals between the grain boundary. (**d**) The growth of crystals blocks the grain boundary, creating a semi-closed system inside the stone. In this semi-closed system, the supersaturation of calcium oxalate is lower compared to the external urine environment, rendering the COD crystals undersaturated and allowing for the nucleation of COM crystals. (**e**) The presence of more thermodynamically stable COM crystals inside the stone causes dissolution of the internal COD crystals. (**f**) Continuous nucleation of COM crystals, thereby progressing the phase transition

The urinary environment is always highly supersaturated for COM and COD crystals [36, 37]. If supersaturation is maintained, it should be difficult for COD crystals to undergo a solution-mediated phase transition to COM crystals because they do not dissolve. Still, the results of this study showed a phase transition from inside of the COD stones to COM crystals. Therefore, it is necessary to consider where COD crystals can dissolve within the body. Generally, the center area of a stone is isolated from urine. When grain boundaries or cracks connect the inside of the stones to the urinary environment, urine can enter the stones (Fig. 4a). The supersaturated solution that penetrating these grain boundaries promotes the nucleation of crystals (either COD or COM) between these boundaries (Fig. 4b). Subsequently, the nucleated crystals grow in the penetrated solution. Over time, these growing crystals enclose grain boundaries, creating a barrier that isolates the inside of the stone from the urinary (Fig. 4c). Thus, the inside of the stone assumes an almost enclosed state, in other words a semi-closed system. Although solution exist in the semi-closed system, the solutes (comprising calcium ions and oxalate ions) are consumed by the growth of crystals between grain boundaries, resulting in a lower solute concentration. Consequently, the solution inside the stone becomes undersaturated for COD crystals. Only COM crystals can nucleate or grow within the semi-closed system (Fig. 4d). As COM crystals grow, a localized undersaturated environment for COD crystals is formed, which, as a result, dissolves the interior COD crystals (Fig. 4e). This dissolution of COD crystals induces the growth of COM crystals (Fig. 4f). Furthermore, the water released during the phase transition (from COD to COM crystals) decreases the calcium oxalate concentration inside of the stone, accelerating this process. Note that such solution-mediated phase transition proceeds gradually through very minute amounts of the solution phase. If stones have more grain boundaries, such semi-closed systems effectively form, and the phase transition progresses preferentially. Sivaguru et al. reported that during the phase transition from COD to COM crystals, voids, which are crystal defects, are formed inside the stone due to the loss of water and decrease in volume [19]. The formation of voids can be a new path connecting the inside of the stone to the urine, an element of further continuous progression of the phase transition. As phase transition advances, mosaic COM structure form.

Solution-mediated phase transition stops in an environment where the COD crystals are constantly supersaturated. The boundary condition maintains a balance between solute consumption and supply near the interface where the COD crystals are in contact with urine. Therefore, COD crystals remain in the periphery area of the stone, surrounding the COM crystals.

In addition to grain boundaries, we believe that inclusion is also related to the progression of the phase transition. An inclusion is solution and/or crystals that are trapped inside a mother crystal during its formation, and it is thermodynamically unstable; thus, such defects will be good starting points for phase transition [38, 39]. Once COM crystals, the stable phase, nucleate from the solution entrapped within inclusions, as previously described, the progression of phase transition proceeds rapidly.

It is hypothesized that stone formation rates increase when the total amount of urine in a patient’s body is small, such as in summer or at night. The faster the crystal grows, the more inclusions develop in the crystal [38]. Therefore, the quality of COD crystals is highly dependent on the patient’s internal environment (season, time of day, etc.), creating regions of high and low density of inclusions [9]. The inclusion distribution probably caused a heterogeneous phase transition in the kidney stone.

### Variations in crystal size

Here, we will mention why the crystals that makeup kidney stones vary so widely in size. The euhedral CODs that initially composed the kidney stone nucleated and grew in the renal pelvis. Because they are constantly exposed to high supersaturated conditions of urine and aggregate after growing in a place with no spatial constraints, there is a wide variation in crystal size, ranging from a few micrometers to millimeter-order, as seen in this study.

Mosaic COM crystals formed in phase transitions were observed with grain sizes ranging from a few micrometers to tens of micrometers, which is large for COM crystals. In a semi-closed system, COM nucleation proceeds slowly in an environment close to equilibrium conditions. Because they coexist and grow together, the number of crystals becomes small, and each develops relatively large. The semi-closed system makes COM crystals snarled because of the spatial constraints. In contrast, the COM crystals that make up concentric COMs, observed in detail in papers [40] and [41], are aggregates of nanometer to semi-micrometer order crystals. Concentric COM is a primary structure that nucleates and grows directly without undergoing a phase transition [9]. We have also recently shown that where calcium phosphate (CaP) crystals are present, COM crystals preferentially nucleate rather than COD crystals, and grow directly on CaP [42]. As many COMs nucleate and grow on the CaP surface, the size of each crystal becomes small due to the competition of solute. On the other hand, since they elongate and grow toward a wide space to form spherulite, faceted shapes are observed in the crystals’ elongation direction (*c*-axis direction). Thus, the process by which the microstructures comprising the stone are formed significantly affects the size and shape of the crystals.

### Phase transition hardens kidney stones

**Fig. 5:**
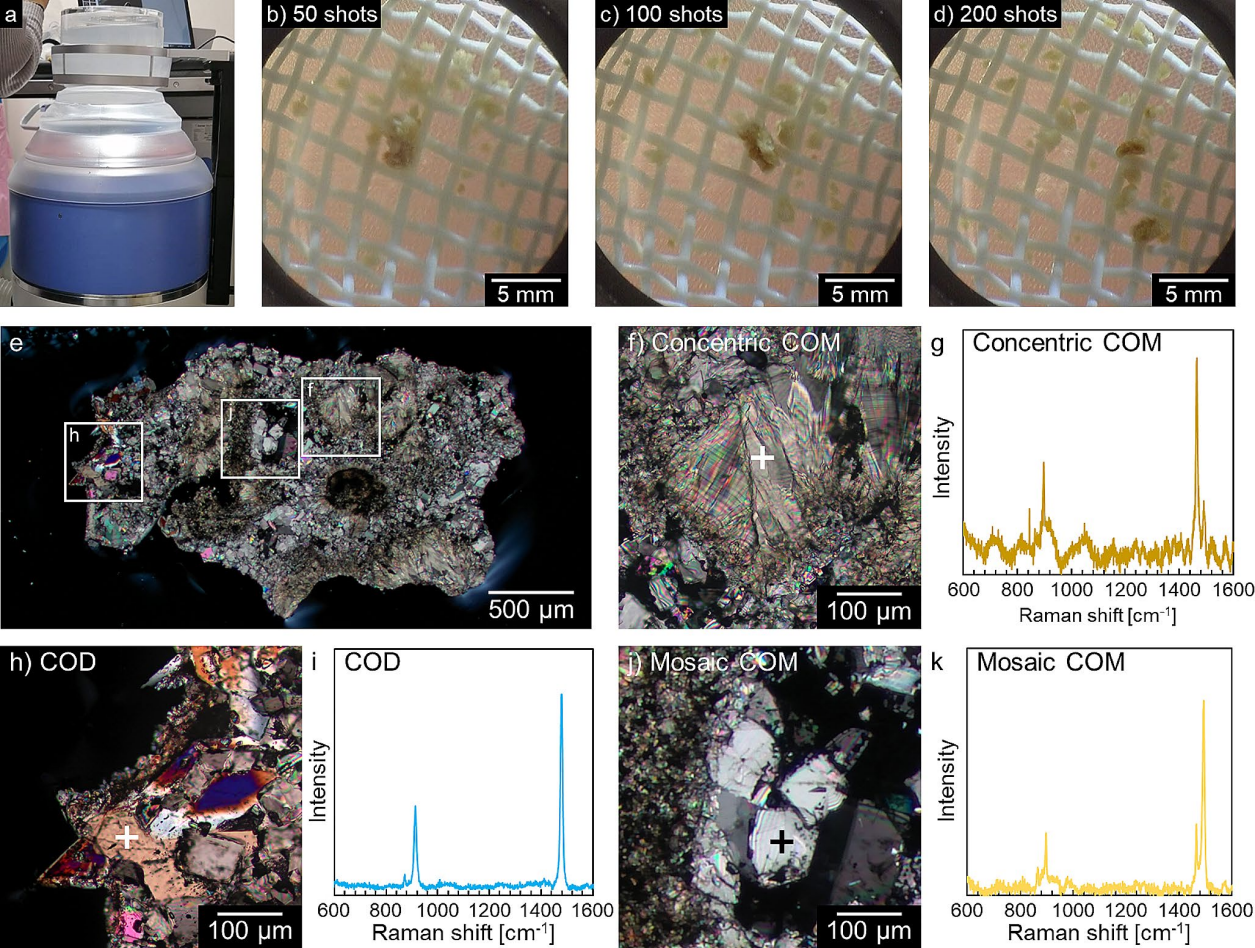
Investigation of the stone (Sample 2)﻿ hardness by a medical extracorporeal shock wave lithotripter. (**a**) Experimental system for crushing stone. A water tank was placed above the device, and stones were placed in a cage attached to the top of the tank and irradiated with shock waves. (**b**) Stone crushing process. The 50th shock wave irradiation. (**c**) The 100th shock wave irradiation. (**d**)The 200th shock wave irradiation. (**e**) Cross-Nicol image of the largest volumetric fragment of the crushed fragments. (**f**) An enlarged cross-Nicol image of Concentric COM region. (**g**) Raman spectra obtained in concentric COM region. (**h**) An enlarged cross-Nicol image of COD region. (**i**) Raman spectra obtained in COD region. (**j**) An enlarged cross-Nicol image of mosaic COM region. (**k**) Raman spectra obtained in mosaic COM region. Only a few COD areas were observed. Most of the crushed fragments consisted of concentric or mosaic COM

To investigate whether these phase-transformed stones would be a problem in the medical field, we evaluated on the hardness of kidney stones and conducted stone-crushing experiments using a medical extracorporeal shock wave lithotripter (ESWL) (Fig. 5a, Supplementary Fig. 4a-c). When the stones were repeatedly subjected to shock waves, the brittle parts of the stones were quickly crushed into smaller pieces, but the hard parts remained as relatively large fragments (Fig. 5b, c, d). When we examined the phase and microstructure of these fragments, we found that most of them consisted of concentric COM structures or densely agglomerated mosaic COM structures (Fig. 5e-k). It means that the mosaic COM structure, composed after the phase transition, is rigid and difficult to crush, just like the concentric COM structure [43]. As the phase transition progresses, the stones gradually become harder and more difficult to crush in stone treatment.

For example, crustaceans demonstrate a remarkable ability to rapidly construct robust hard tissues by effectively controlling the assembly of amorphous or metastable phases [1–3]. This controlled phase transition serves as an intelligent survival strategy for these organisms. In ironic contrast, the process by which the organism endeavors to build harder structures exacerbates the formation of kidney stones, leading to detrimental consequences.

### What controls the phase transition rate

We observed the formation of thin, shell-like COM crystals on the surface of the stone. Such structures are not commonly observed in stones that form in the human body. The reason why the structure was created in this experiment can be attributed to the experimental conditions.

At the start of the experiment, the solution was supersaturated for COD phase. However, as phase transition inside the stone progressed, the overall concentration of the solution decreased (Supplementary Fig. 3). It is reasonable to consider that this temporary decrease in solution concentration caused the phase transition at the outermost surface. If the solution had been maintained at high levels of supersaturation, as in real urine, phase transition would have predominantly proceeded in the center regions of the stone. The observed phase transition rate inside the stone averaged several tens of micrometers per day, between the start and two weeks after the experiment. In a patient’s body, the phase transition rate of COD stones is not precisely known but COD crystal components in kidney stones often persist for several months to years. Comparing the fact and our experimental results, the phase transition rate (several tens of micrometers per day) is notably fast. The implication drawn from these findings is that a reduction in the calcium oxalate supersaturation in urine results in a significantly accelerated phase transition. When evidence of stone dissolution within the body (specifically, COD crystals) was reported, it led to affirmative discussions regarding the potential for in vivo stone dissolution therapy [19]. However, in reality, in an environment where stones dissolve, denser, harder, and more troublesome COM stones are newly formed, exacerbating the condition. Therefore, while lowering urine supersaturation reduces nucleation and growth of new crystals, it may cause stone disease more refractory by expediting phase transition. This supports the necessity of early intervention in stone treatment.

## Conclusion

In this study, we succeeded in observing the phase transition process of the stone, which are mostly composed of COD crystals. Detailed analysis of the stone revealed that the phase transition of COD stones is triggered by crystal defects such as grain boundaries. The COM stone that develop after the phase transition are harder and more difficult to crush than COD stone, suggesting that the phase transition is a factor that aggravates the disease. One of the methods of stone treatment and prevention is drinking plenty of water, but the decrease in urinary calcium oxalate supersaturation caused by this drinking may accelerate the progression of phase transitions, thereby making the stones more malignant. Thus, the follow-up of lithiasis leads to stone hardening, i.e., worsening of the stones in the body. The findings of this study strongly support the need for early treatment in clinical practice.

## Electronic supplementary material

Below is the link to the electronic supplementary material.

Supplementary Material 1

Supplementary Material 2

Supplementary Material 3

Supplementary Material 4

Supplementary Material 5

Supplementary Material 6

Supplementary Material 7

Supplementary Material 8

## Data Availability

Data is provided within the manuscript or supplementary information files.Our manuscript is on Research Square.https://doi.org/10.21203/rs.3.rs-3687835/v1.

## References

[1] LeGeros RZ (2001) Formation and transformation of calcium phosphates: relevance to vascular calcification. Z Kardiol 90:116–124. 10.1007/s003920170032 11374023 10.1007/s003920170032

[2] Gong N, Shangguan J, Liu X et al (2008) Immunolocalization of matrix proteins in nacre lamellae and their in vivo effects on aragonitic tablet growth. J Struct Biol 164:33–40. 10.1016/j.jsb.2008.05.009 18620869 10.1016/j.jsb.2008.05.009

[3] Veis A, Dorvee JR (2013) Biomineralization mechanisms: a new paradigm for crystal nucleation in organic matrices. Calcif Tissue Int 93:307–315. 10.1007/s00223-012-9678-2 23241924 10.1007/s00223-012-9678-2 PMC3726565

[4] Yamashita I, Hayashi J, Hara M (2004) Bio-template synthesis of uniform CdSe nanoparticles using cage-shaped protein, apoferritin. Chem Lett 33:1158–1159. 10.1246/cl.2004.1158

[5] Sugawara A, Nishimura T, Yamamoto Y et al (2006) Self-organization of oriented calcium carbonate/polymer composites: effects of a matrix peptide isolated from the exoskeleton of a crayfish. Angew Chemie - Int Ed 45:2876–2879. 10.1002/anie.200503800 10.1002/anie.200503800 16550616

[6] Khan SR, Pearle MS, Robertson WG et al (2016) Kidney stones. Nat Rev Dis Prim 2:16008–16057. 10.1038/nrdp.2016.8 27188687 10.1038/nrdp.2016.8 PMC5685519

[7] Sivaguru M, Saw JJ, Wilson EM et al (2021) Human kidney stones: a natural record of universal biomineralization. Nat Rev Urol 18:404–432. 10.1038/s41585-021-00469-x 34031587 10.1038/s41585-021-00469-x

[8] Tostivint IN, Araman RG, Castiglione V et al (2022) How the diagnosis and the management of genetic renal phosphate leak impact the life of kidney stone formers? Urolithiasis 50:319–331. 10.1007/s00240-022-01316-3 35224662 10.1007/s00240-022-01316-3

[9] Maruyama M, Tanaka Y, Momma K et al (2023) Evidence for solution-mediated phase transitions in kidney stones: Phase Transition exacerbates kidney Stone Disease. Cryst Growth Des 23:4285–4293. 10.1021/acs.cgd.3c00108

[10] Pak CYC (1998) Kidney stones. Lancet 351:1797–1801. 10.1016/S0140-6736(98)01295-1 9635968 10.1016/S0140-6736(98)01295-1

[11] Romero V, Akpinar H, Assimos DG (2010) Kidney stones: a global picture of prevalence, incidence, and associated risk factors. Rev Urol 12:e86–96. 10.3909/riu0459 20811557 PMC2931286

[12] Saitoh H, DESCRIPTIONS OF URINE IN THE HIPPOCRATIC COLLECTION (2006) Japanese J Urol 97:10–19. 10.5980/jpnjurol1989.97.10 10.5980/jpnjurol1989.97.10 16485549

[13] Boyce WH (1968) Organic matrix of human urinary concretions. Am J Med 45:673–683. 10.1016/0002-9343(68)90203-9 5687257 10.1016/0002-9343(68)90203-9

[14] Warpehoski MA, Buscemi PJ, Osborn DC et al (1981) Distribution of organic matrix in calcium oxalate renal calculi. Calcif Tissue Int 33:211–222. 10.1007/BF02409440 6791784 10.1007/BF02409440

[15] Coe FL, Evan A, Worcester E (2005) Science in medicine kidney stone disease. J Clin Investig 115:2598–2608. 10.1172/JCI26662.2598 16200192 10.1172/JCI26662 PMC1236703

[16] Khan SR (2006) Renal tubular damage/dysfunction: key to the formation of kidney stones. Urol Res 34:86–91. 10.1007/s00240-005-0016-2 16404622 10.1007/s00240-005-0016-2

[17] Basavaraj DR, Biyani CS, Browning AJ, Cartledge JJ (2007) The role of urinary kidney stone inhibitors and promoters in the pathogenesis of Calcium containing Renal stones. EAU-EBU Updat Ser 5:126–136. 10.1016/j.eeus.2007.03.002

[18] Alelign T, Petros B (2018) Kidney Stone Disease: an update on current concepts. 10.1155/2018/3068365. Adv Urol 2018:10.1155/2018/3068365 PMC5817324 29515627

[19] Sivaguru M, Saw JJ, Williams JC et al (2018) Geobiology reveals how human kidney stones dissolve in vivo. Sci Rep 8:13731. 10.1038/s41598-018-31890-9 30213974 10.1038/s41598-018-31890-9 PMC6137216

[20] Grases F, Costa-Bauzá A, García-Ferragut L (1998) Biopathological crystallization: a general view about the mechanisms of renal stone formation. Adv Colloid Interface Sci 74:169–194. 10.1016/S0001-8686(97)00041-9 9561720 10.1016/s0001-8686(97)00041-9

[21] Maruyama M, Sawada KP, Tanaka Y et al (2023) Quantitative analysis of calcium oxalate monohydrate and dihydrate for elucidating the formation mechanism of calcium oxalate kidney stones. PLoS ONE 18:1–15. 10.1371/journal.pone.0282743 10.1371/journal.pone.0282743 PMC9997882 36893192

[22] Heijnen WMM, Van Duijneveldt FB (1984) The theoretical growth morphology of calcium oxalate dihydrate. J Cryst Growth 67:324–336. 10.1016/0022-0248(84)90192-1

[23] Chien YC, Masica DL, Gray JJ et al (2009) Modulation of calcium oxalate dihydrate growth by selective crystal-face binding of phosphorylated osteopontin and polyaspartate peptide showing occlusion by sectoral (compositional) zoning. J Biol Chem 284:23491–23501. 10.1074/jbc.M109.021899 19581305 10.1074/jbc.M109.021899 PMC2749123

[24] Momma K, Izumi F (2011) VESTA 3 for three-dimensional visualization of crystal, volumetric and morphology data. J Appl Crystallogr 44:1272–1276. 10.1107/S0021889811038970

[25] Zarse CA, McAteer JA, Sommer AJ et al (2004) Nondestructive analysis of urinary calculi using micro computed tomography. BMC Urol 4:1–8. 10.1186/1471-2490-4-15 15596006 10.1186/1471-2490-4-15 PMC544194

[26] Tajiri R, Fujita T (2013) Observation methods of hard and soft animal tissues by grinding specimens impregnated with resin. Taxa. Proc Japan Soc Syst Zool 35:24–34. 10.19004/taxa.35.0_24

[27] Selvaraju R, Raja A, Thiruppathi G (2012) FT-Raman spectral analysis of human urinary stones. Spectrochim Acta - Part Mol Biomol Spectrosc 99:205–210. 10.1016/j.saa.2012.09.004 10.1016/j.saa.2012.09.004 23069621

[28] Tanaka Y, Maruyama M, Okada A et al (2021) Multicolor imaging of calcium – binding proteins in human kidney stones for elucidating the effects of proteins on crystal growth. Sci Rep 11:16841–16841. 10.1038/s41598-021-95782-1 34446727 10.1038/s41598-021-95782-1 PMC8390759

[29] Khan SR, Hackett RL (1986) Identification of urinary stone and sediment crystals by scanning electron microscopy and X-ray microanalysis. J Urol 135:818–825. 10.1016/S0022-5347(17)45868-X 3959214 10.1016/s0022-5347(17)45868-x

[30] Cloutier J, Villa L, Traxer O, Daudon M (2015) Kidney stone analysis: give me your stone, I will tell you who you are! World J Urol 33:157–169. 10.1007/s00345-014-1444-9 25465911 10.1007/s00345-014-1444-9 PMC4308647

[31] Bazin D, Leroy C, Tielens F et al (2016) Hyperoxaluria is related to whewellite and hypercalciuria to weddellite: what happens when crystalline conversion occurs? Comptes Rendus Chim 19:1492–1503. 10.1016/j.crci.2015.12.011

[32] Jacob DE, Grohe B, Geßner M et al (2013) Kidney stones in primary hyperoxaluria: New lessons Learnt. PLoS ONE 8:e70617–70625. 10.1371/journal.pone.0070617 23940605 10.1371/journal.pone.0070617 PMC3734250

[33] Williams JC, Worcester E, Lingeman JE (2017) What can the microstructure of stones tell us? Urolithiasis 45:19–25. 10.1007/s00240-016-0944-z 27913855 10.1007/s00240-016-0944-z PMC5253090

[34] Williams JC, Lingeman JE, Daudon M, Bazin D (2022) Using micro computed tomographic imaging for analyzing kidney stones. Comptes Rendus Chim 25:61–72. 10.5802/crchim.89 10.5802/crchim.89 PMC8312732 34321982

[35] Momma K, Ikeda T, Nishikubo K et al (2011) New silica clathrate minerals that are isostructural with natural gas hydrates. Nat Commun 2:196–202. 10.1038/ncomms1196 21326228 10.1038/ncomms1196

[36] Streit J, Tran-Ho LC, Königsberger E (1998) Solubility of the three calcium oxalate hydrates in sodium chloride solutions and urine-like liquors. Monatshefte fur Chemie 129:1225–1236. 10.1007/PL00010134

[37] Baumann JM, Casella R (2019) Prevention of Calcium Nephrolithiasis: the influence of Diuresis on Calcium Oxalate crystallization in urine. Adv Prev Med 2019:1–8. 10.1155/2019/3234867 10.1155/2019/3234867 PMC6448328 31016047

[38] Ivan VM (2003) Crystal Growth for beginners: fundamentals of Nucleation, Crystal Growth, and Epitaxy, 2nd edn. World Scientific

[39] Maruyama M, Yoshikawa HY, Takano K et al (2023) Solution-mediated phase transition of pharmaceutical compounds: case studies of acetaminophen and aspirin. J Cryst Growth 602:126990–126998. 10.1016/j.jcrysgro.2022.126990

[40] Dorian HH, Rez P, Drach GW (1996) Evidence for aggregation in Oxalate Stone formation: Atomic Force and low voltage scanning Electron Microscopy. J Urol 156:1833–1837. 10.1016/S0022-5347(01)65547-2 8863626 10.1016/s0022-5347(01)65547-2

[41] Sandersius S, Rez P (2007) Morphology of crystals in calcium oxalate monohydrate kidney stones. Urol Res 35:287–293. 10.1007/s00240-007-0115-3 17899050 10.1007/s00240-007-0115-3

[42] Michibata U, Maruyama M, Tanaka Y et al (2024) Calcium phosphate controls nucleation and growth of calcium oxalate crystal phases in kidney stones. Biomed Res Febr 20th 2024 accepted 10.2220/biomedres.45.103 38839353

[43] Heimbach D, Munver R, Zhong P et al (2000) Acoustic and mechanical properties of artificial stones in comparison to natural kidney stones. J Urol 164:537–544. 10.1016/S0022-5347(05)67419-8 10893640

