# Supplementary figures and images for: The impact of crystal phase transition on the hardness and structure of kidney stones
Source: Urolithiasis. 2024 Apr 2;52(1):57. doi: 10.1007/s00240-024-01556-5 (PMC10987347; doi:10.1007/s00240-024-01556-5)

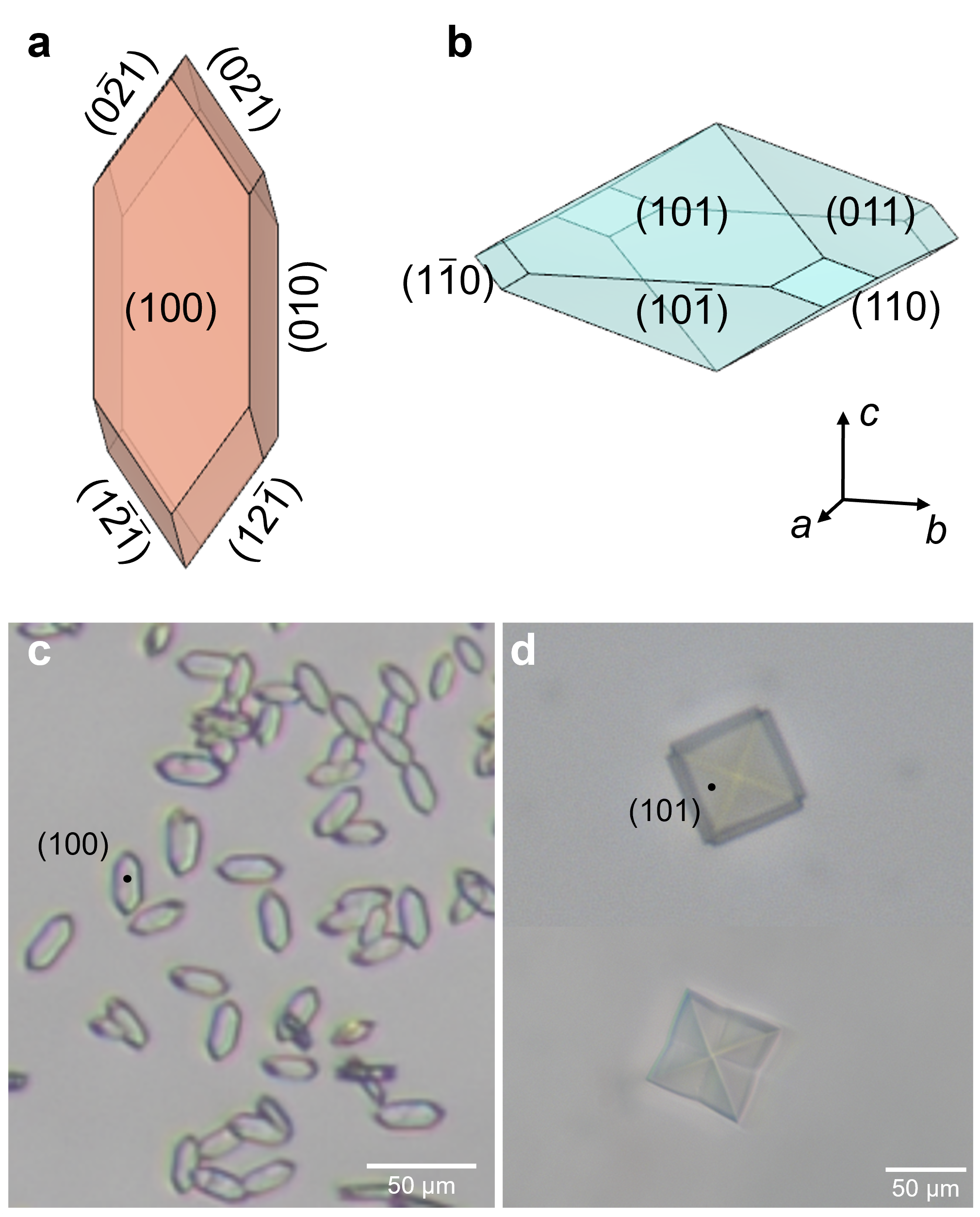

Supplement: Supplementary file 1 — Supplementary Material 1 [file 240_2024_1556_MOESM1_ESM.tif]

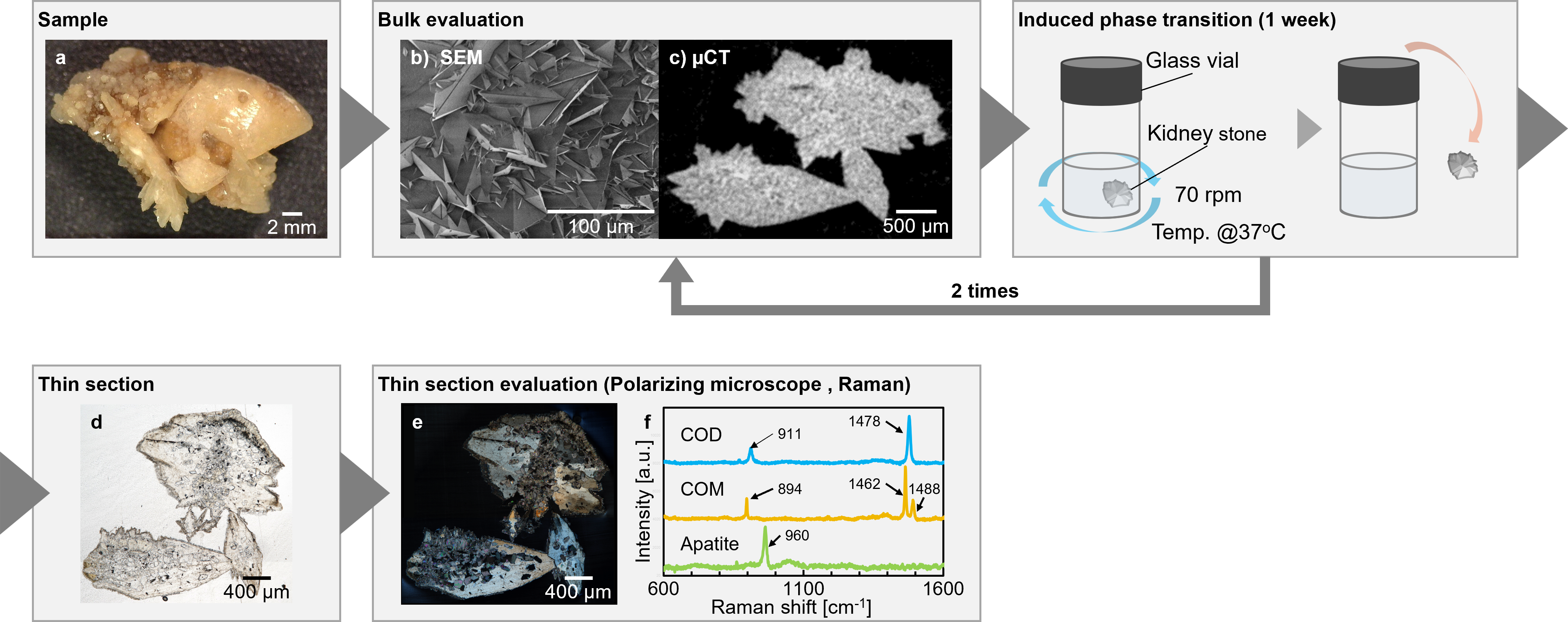

Supplement: Supplementary file 2 — Supplementary Material 2 [file 240_2024_1556_MOESM2_ESM.tif]

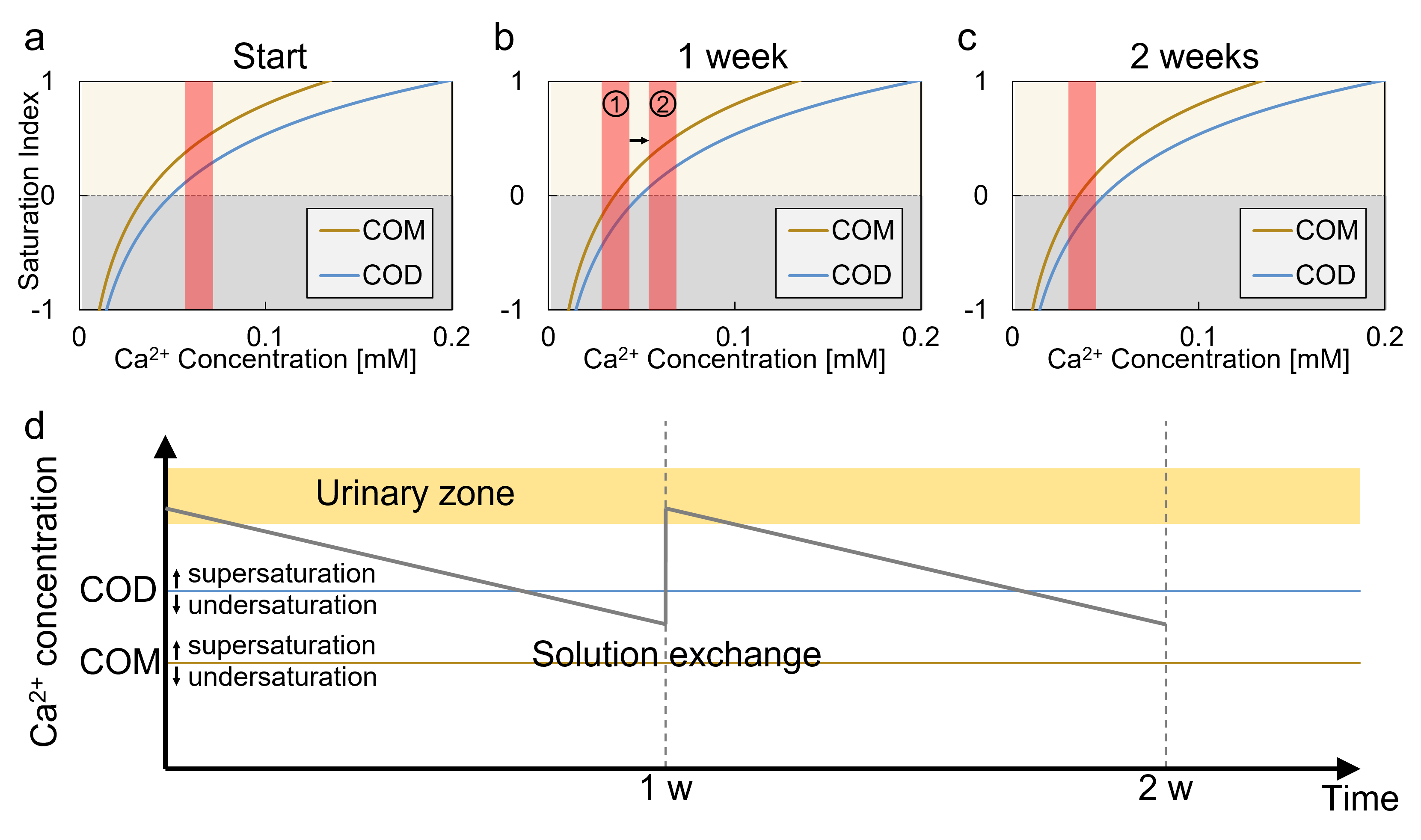

Supplement: Supplementary file 3 — Supplementary Material 3 [file 240_2024_1556_MOESM3_ESM.tif]

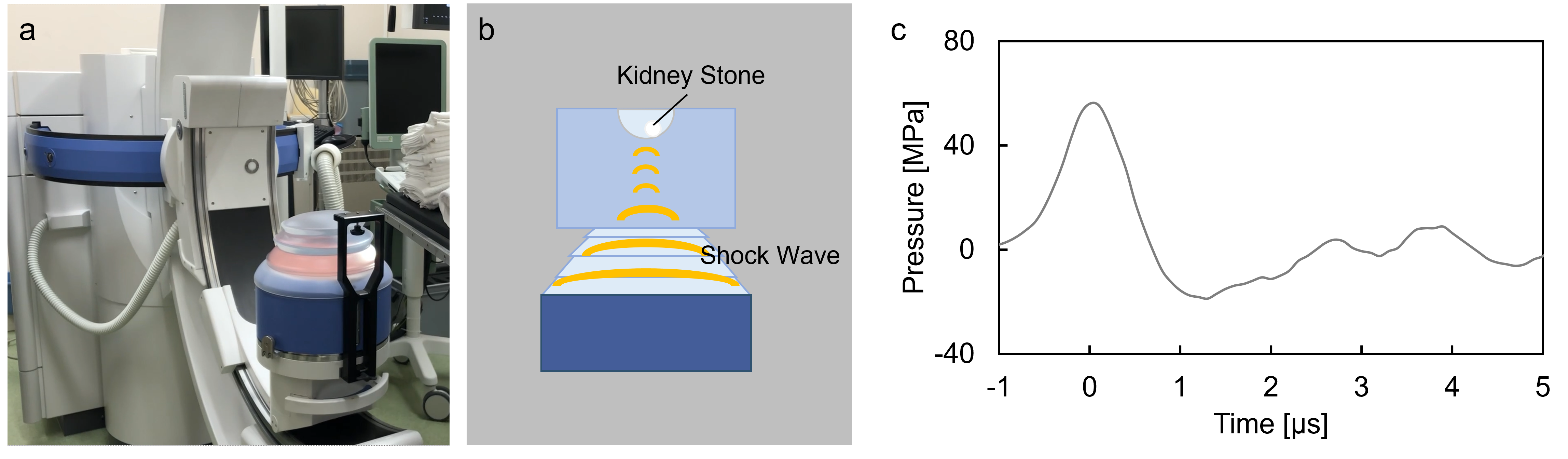

Supplement: Supplementary file 4 — Supplementary Material 4 [file 240_2024_1556_MOESM4_ESM.tif]

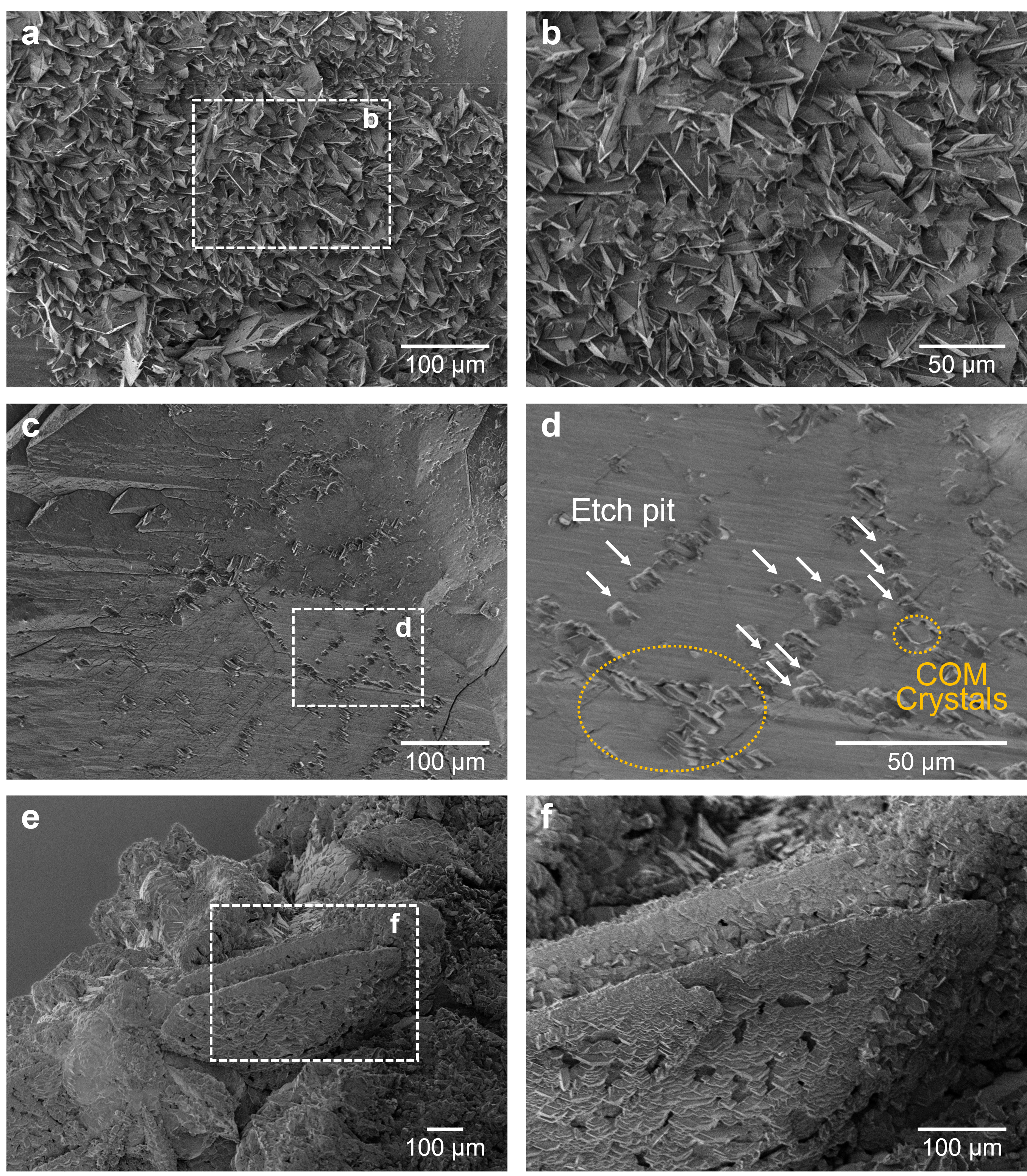

Supplement: Supplementary file 5 — Supplementary Material 5 [file 240_2024_1556_MOESM5_ESM.tif]

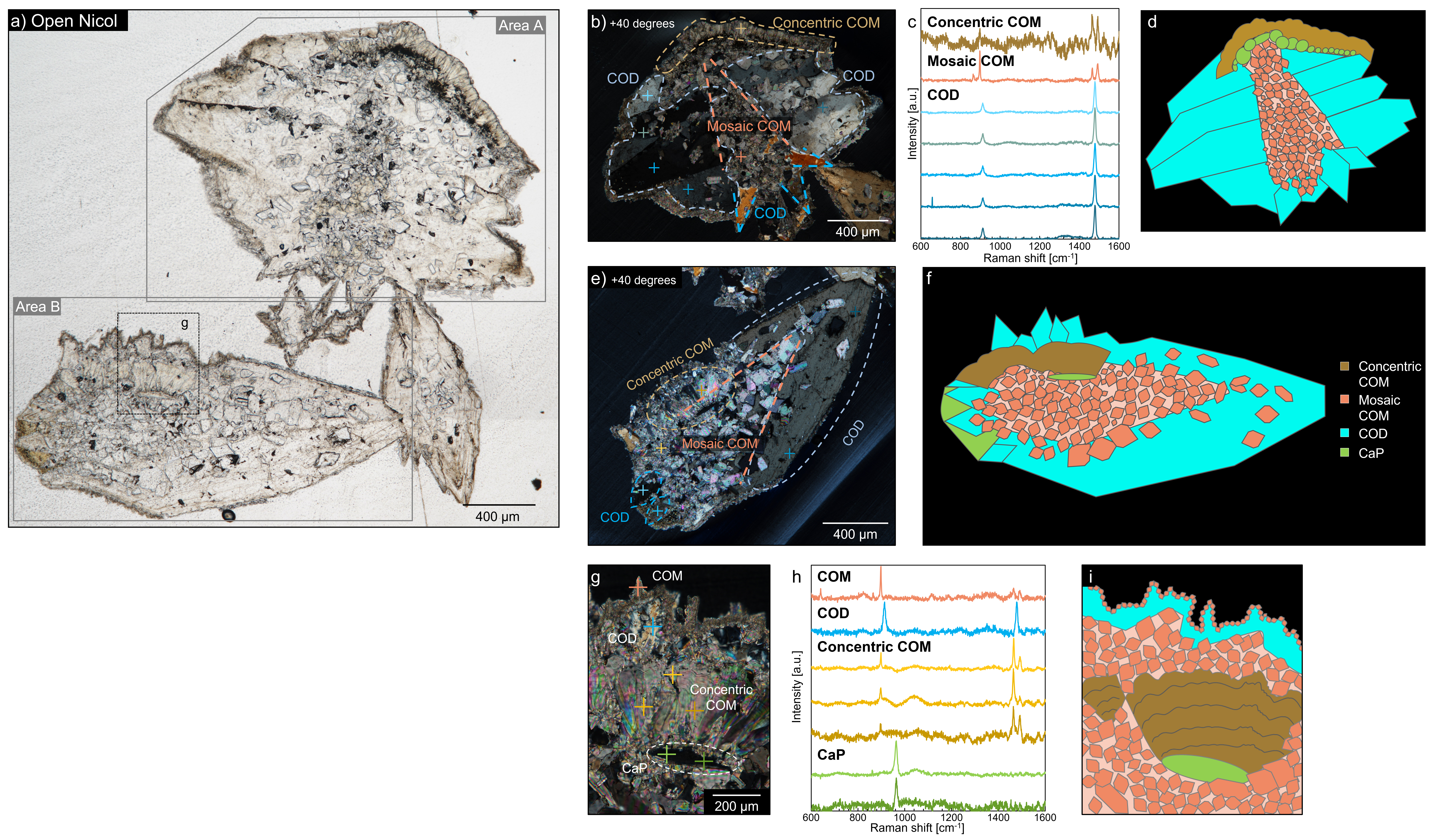

Supplement: Supplementary file 6 — Supplementary Material 6 [file 240_2024_1556_MOESM6_ESM.tif]

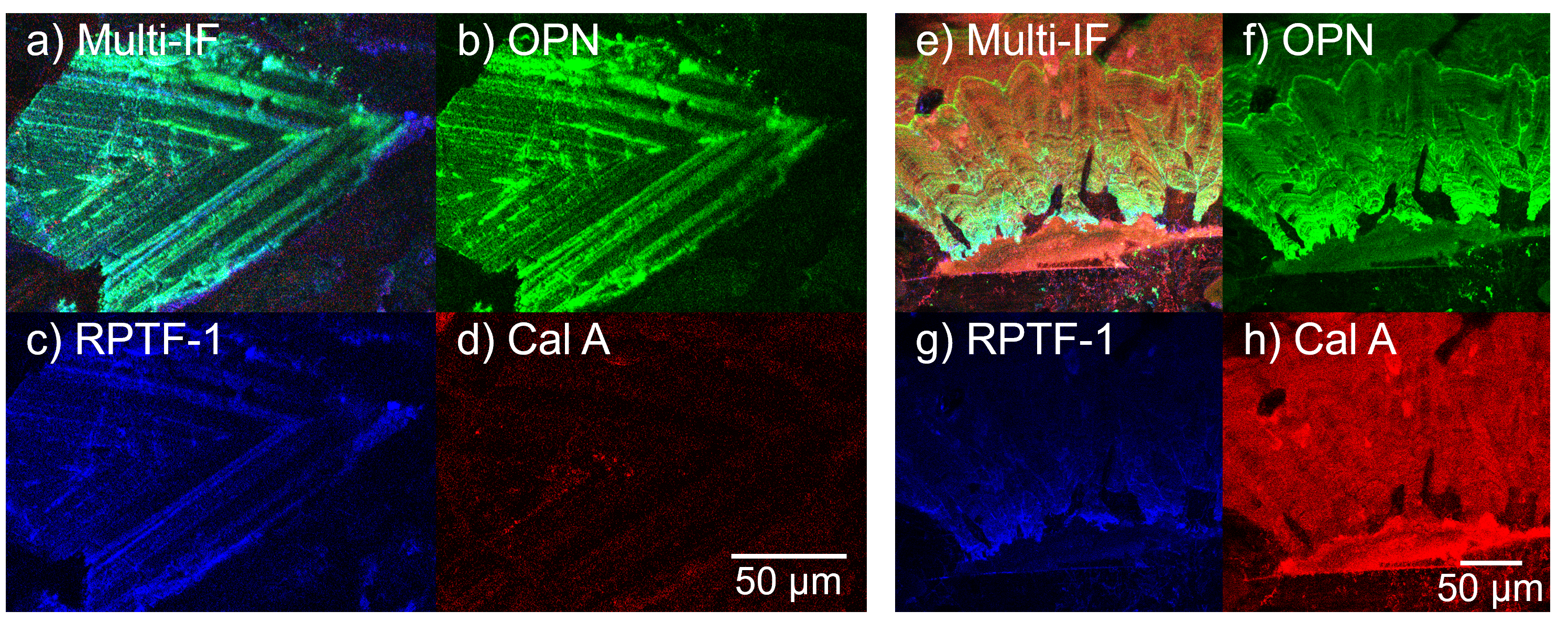

Supplement: Supplementary file 7 — Supplementary Material 7 [file 240_2024_1556_MOESM7_ESM.tif]
