# Supplementary material for: The impact of crystal phase transition on the hardness and structure of kidney stones
Source: Urolithiasis. 2024 Apr 2;52(1):57. doi: 10.1007/s00240-024-01556-5 (PMC10987347; doi:10.1007/s00240-024-01556-5)
Supplement: Supplementary file 8 — Supplementary Material 8 [file 240_2024_1556_MOESM8_ESM.docx]

**
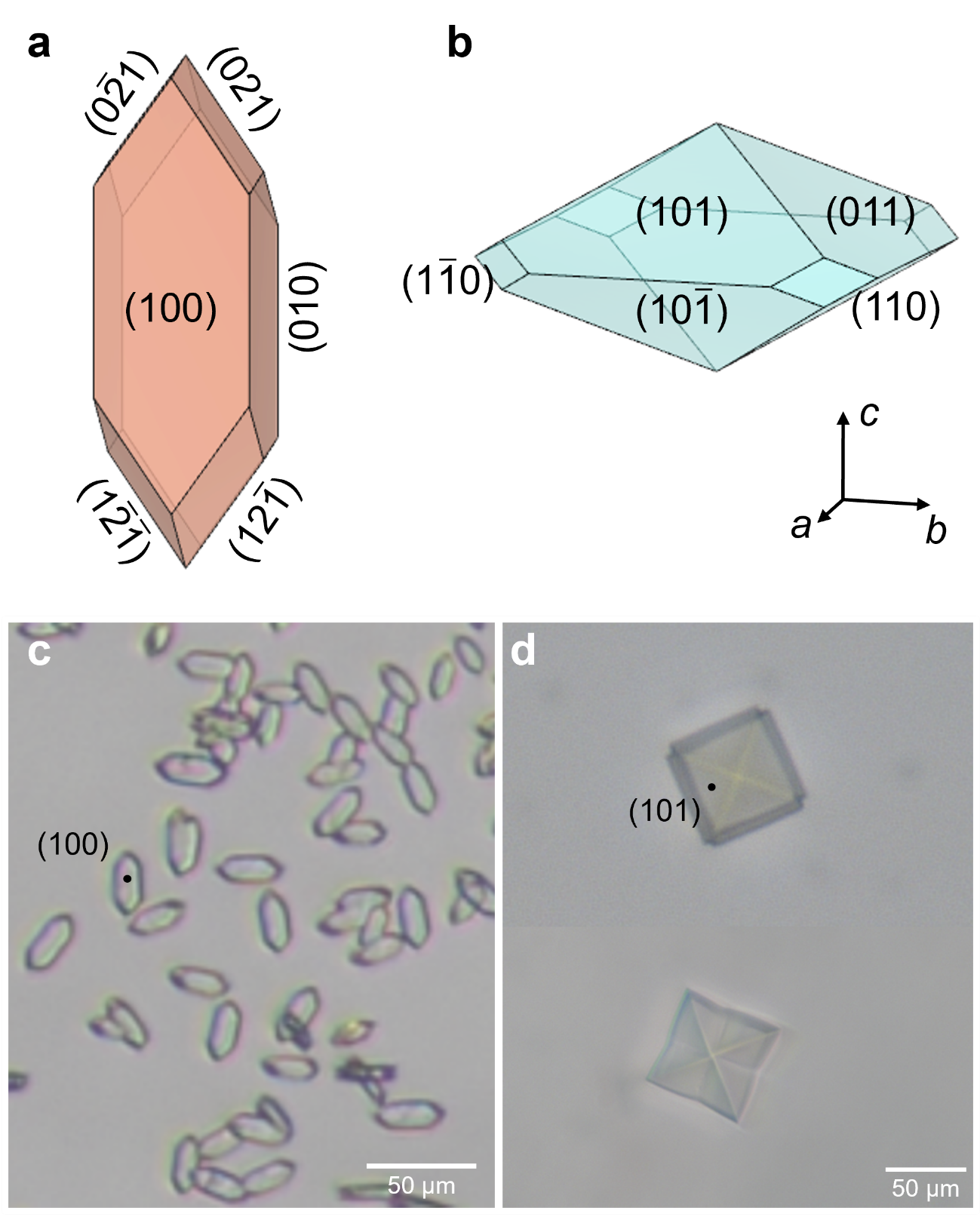
**

Supplementary **Fig.1** COM and COD crystal. (a) A schematic image of a COM crystal. The angle between the {100} and {121} faces is 142°. (b) A schematic image of a COD crystal. The angle between the {101} and {10$\bar{\text{1}}$} faces is 46°. (c) Experimentally obtained COM crystals. (d) Experimentally obtained COD crystals

**
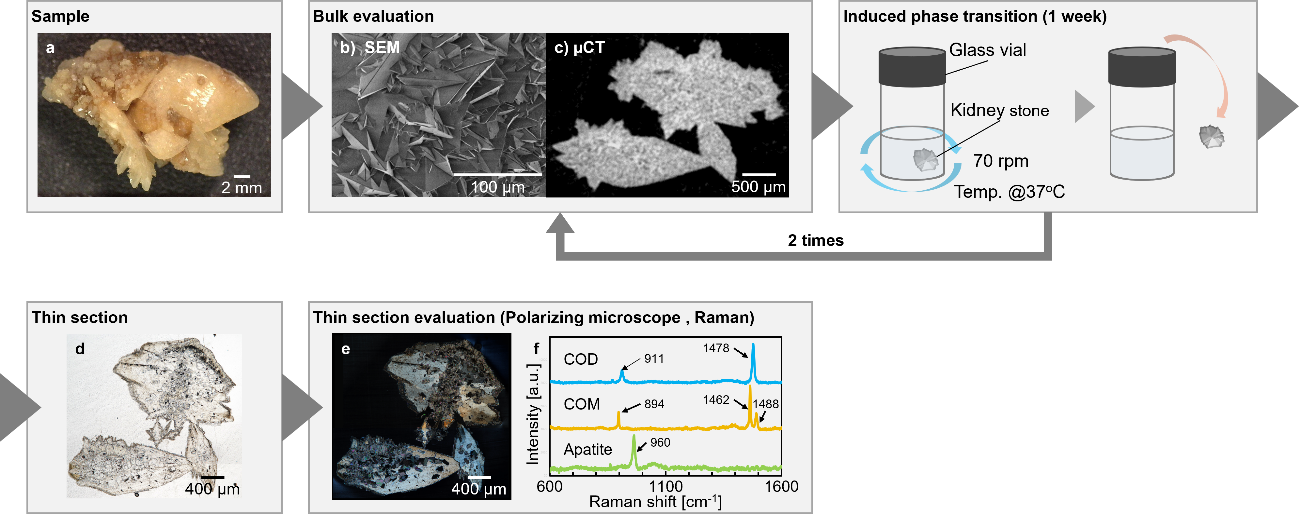
**

Supplementary **Fig.2** Experimental procedure and analysis method. a-e, Flow chart of the experiment. The surface of the COD stone was observed by a digital microscope (a) and SEM (b). The three-dimensional crystal structure inside the stone was observed by X-ray micro-CT (c). The COD stone was placed in a glass vial containing the prepared calcium oxalate solution, and the vial was kept in an incubator. The solution was incubated with gentle shaking at 70 rpm using a rotary shaker. After one week removed from the solution and re-evaluated. This operation was repeated twice. The stone was processed into thin sections with 20-30 µm thickness(d). The microstructures of crystals were observed with a polarizing microscope (e). Further detailed components were identified by Raman spectra (f)


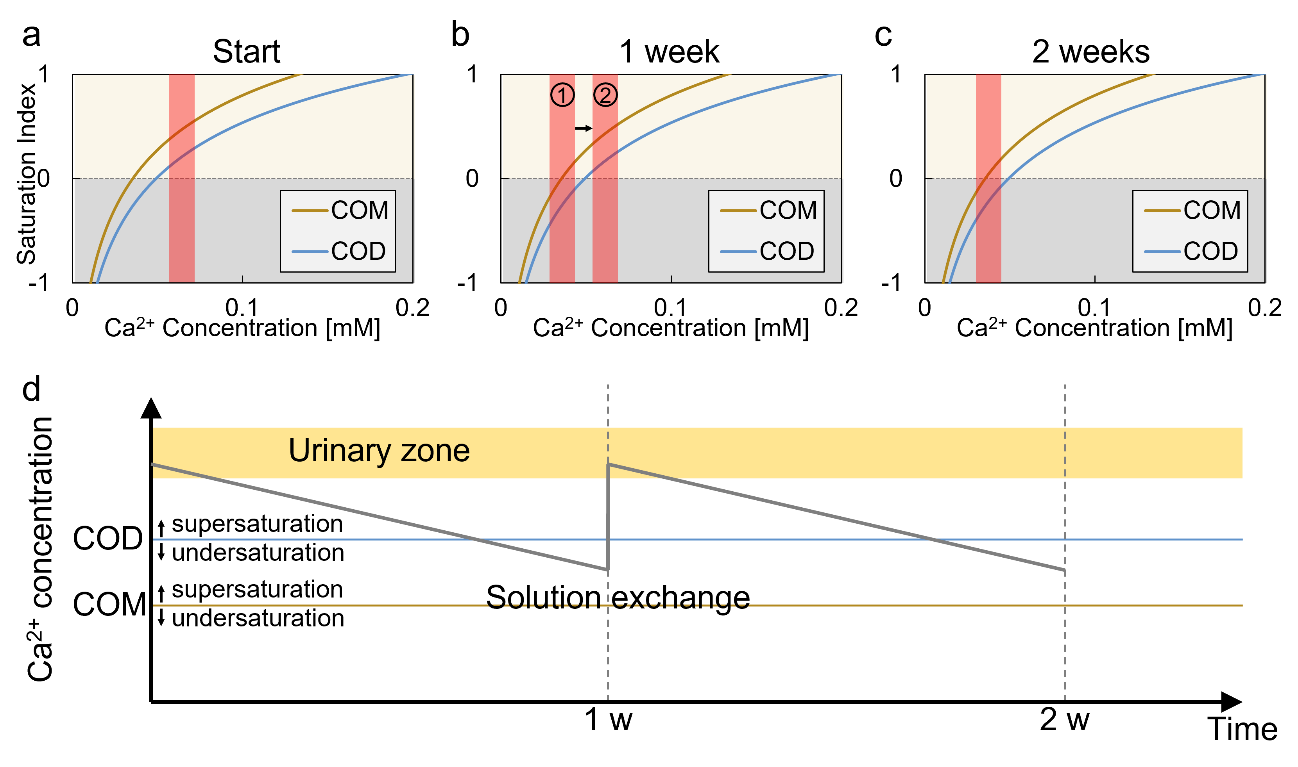
 Supplementary **Fig.3** Estimated changes in calcium ion concentration during the experiment. (a) Saturation indices of COD and COM phases vs the calcium ion concentration at the start of the experiment. The solution was supersaturated for both COD and COM phases (approximate red band Ca^2+^ concentration). (b) Saturation indices of COD and COM phases vs the calcium ion concentration after one week. The solution was undersaturated for COD phase (cercle 1 area). Following solution exchange, it again became supersaturated for both COD and COM phase (cercle 2 area) (c) Saturation indices of COD and COM phases vs the calcium ion concentration after two weeks. The solution once again became undersaturated for COD phase (approximate red band Ca^2+^ concentration). (d) Calcium ion concentration in the solution vs the time during the experiment. Through the experiment, it seemed that the stones were immersed in a solution with calcium ion concentrations significantly lower than those found in the normal urinary environment


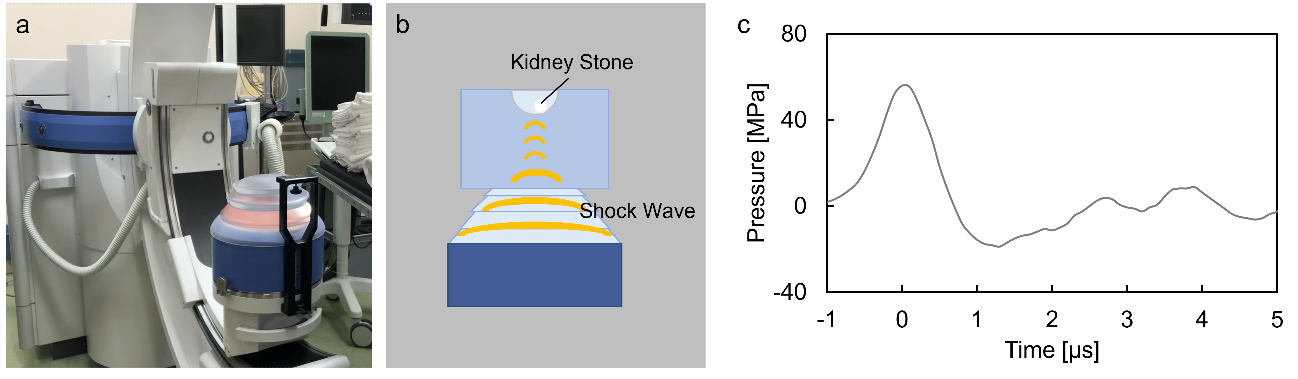


Supplementary **Fig.4** Experimental system for extracorporeal shock wave lithotripsy (ESWL). (a) An extracorporeal shock wave lithotripter (Dornier MedTech, Dornier Gemini). (b) Schematic diagram of the experimental system. (c) Waveform of shock wave given to the COD stone. The peak strength was about 50 MPa

**
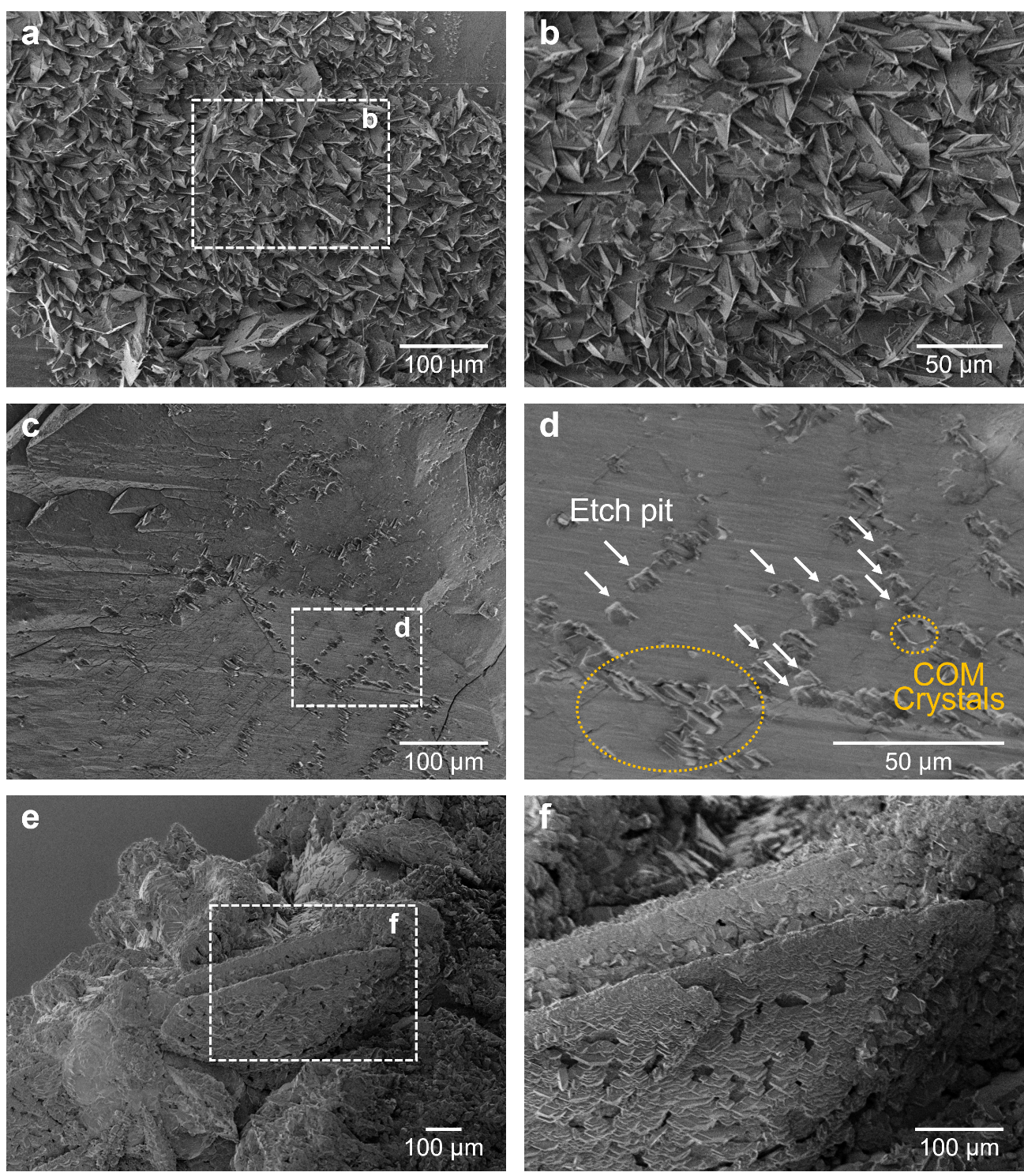
**

Supplementary **Fig.5** COD stone surface observation with SEM. (a) The SEM image of the COD stone at the start of the experiment. The larger sized COD crystal surfaces are covered by smaller sized COD crystals. (b) The enlarged image of the white dotted box area in (a). (c) The SEM image of the COD stone one week after the start of the experiment. Etch-pits formed due to dissolution of the COD crystal surface. At the same time, COM crystals are nucleating. (d) The enlarged image of the white dotted box area in (c). (e) The SEM image of the COD stone two weeks after the start of the experiment. The COD crystals surface was covered with COM crystals. (f) The enlarged image of the white dotted box area in (e)


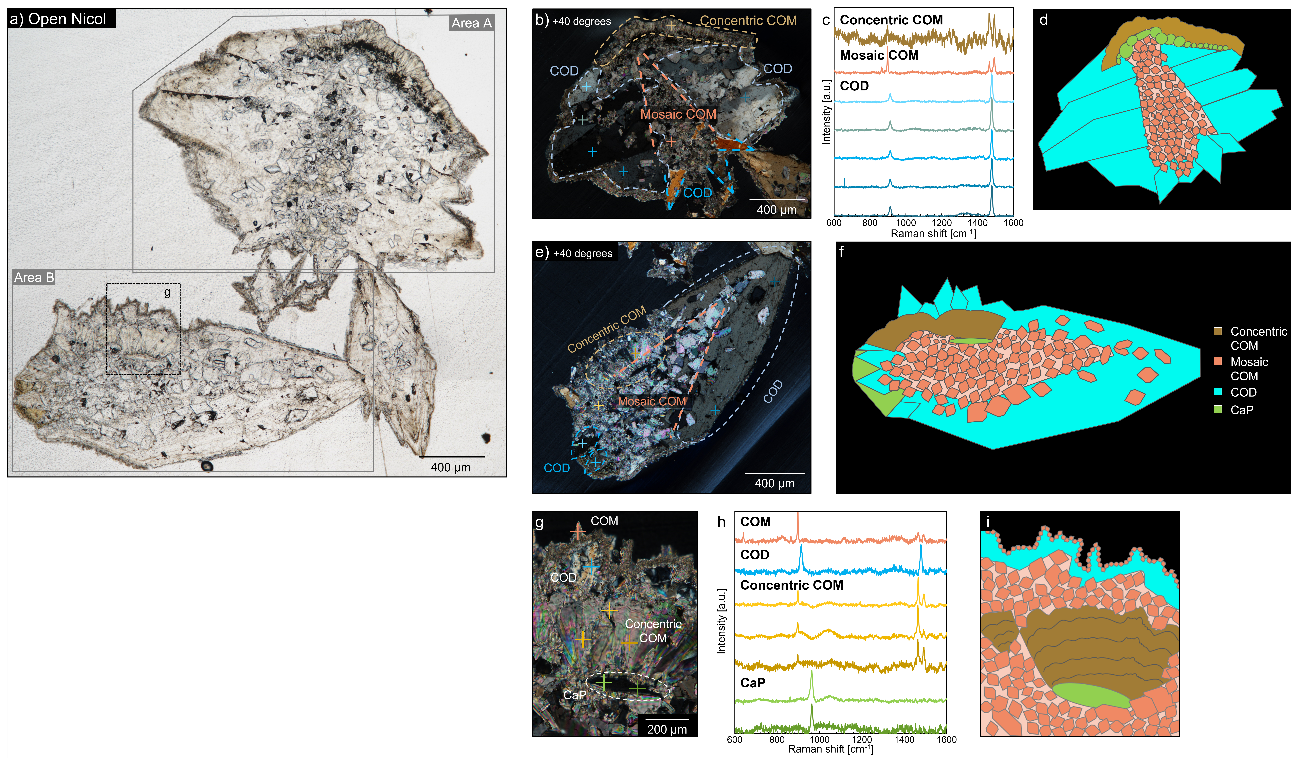


Supplementary **Fig.6** Thin section analysis of the COD stone by a polarizing microscope and Raman spectrometer. (a) Open-Nicol image. (b) An enlarged cross-Nicol image of Area A. The outline of the mosaic COM inside the COD (orange dashed line) is 41.3°, which is similar to the face angle of the COD crystal. (c) The Raman spectra of the cross-hatched area. (d) The mapping of components within Area A. (e) An enlarged cross-Nicol image of Area B. The outline of the mosaic COM inside the COD (orange dashed line) is 40.2°. (f) The mapping of components within Area B. (g) An enlarged cross-Nicol image of the black dotted box area. (h) The Raman spectra of the cross-hatched area. (i) The mapping of components within Area B. The tissue containing microcrystals of CaP found at the base of concentric COM


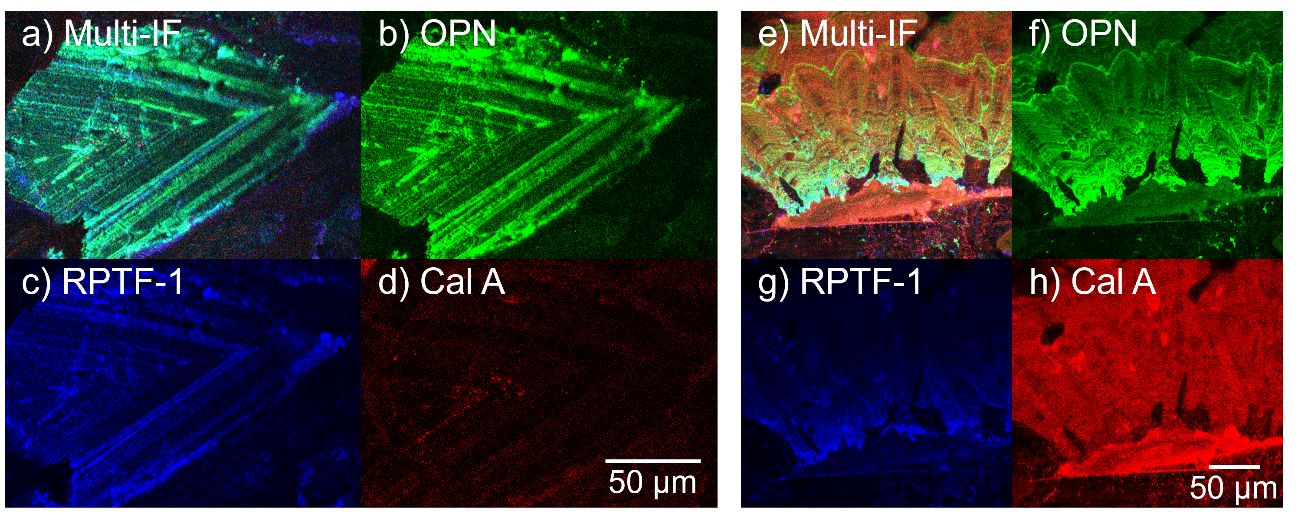


Supplementary **Fig.7** Results of multi-IF staining in COD crystal and concentric COM Structure. (a) Multi-IF staining image of COD crystal. (b) Staining for OPN. c, Staining for RPTF-1. OPN (a) and RPTF-1 (c) striation are distributed in COD crystal. Cal A (d) is present in small quantities. (e) Multi-IF staining image of concentric COM structure. (f) Staining for OPN. (g) Staining for RPTF-1. (h) Staining for Cal A
